# Efficacy and safety of flow diverters for the treatment of intracranial aneurysms: a systematic review and meta-analysis

**DOI:** 10.3389/fsurg.2026.1788189

**Published:** 2026-08-10

**Authors:** Liyong Sun, Chuan He, Ming Ye, Peng Zhang, Yongjie Ma, Jingwei Li, Tongyu Zhang, Xin Guan, Piao Yang, Zhigang Tian, Wenwen Jia, Jianhua Zhang, Hongqi Zhang

**Affiliations:** Department of Neurosurgery, Xuanwu Hospital Capital Medical University, Beijing, China

**Keywords:** efficacy, flow diverters, intracranial aneurysm, meta-analysis, safety

## Abstract

**Objective:**

To examine the efficacy and safety of multiple brands of flow diverters (FDs) in the treatment of intracranial aneurysms (IAs).

**Methods:**

PubMed, EMBASE, and Cochrane were searched for studies on FDs treatment of IAs published until April 11, 2023. The efficacy outcomes were procedural success rate, retreatment, and complete/near-complete occlusion rate. The safety outcomes were postoperative in-stent restenosis (ISR), IA rupture, mortality, cerebrovascular events, and neurogenic death.

**Results:**

104 studies were included. The pooled procedural success rate was 99% (95% CI: 98%–99%). The pooled overall and 1-year retreatment rates were 4% (95% CI: 3%–5%) and 1% (95% CI: 0%–3%), respectively. The pooled complete occlusion and complete/near-complete occlusion rates were 73% and 85% at 6 months, and 80% and 87% at 12 months, respectively. Subgroup analyses suggested p64™ had high 6-month (79%, 95% CI: 72%–84%) and 12 months (91%, 95% CI: 82%–97%) complete occlusion rates and low rescue rates (2%, 95% CI: 0%–6%). The overall aneurysm rupture rate, all-cause mortality, and overall ISR were 1%, 2% and 5%, respectively. The 1-month rates of neurogenic death, hemorrhagic, ischemic, and thromboembolic events were 1%, 2%, 5% and 3%, respectively.

**Conclusion:**

FDs treatment for IA achieves high procedural success and occlusion rates, and the safety profile is favorable with a low incidence of IA rupture and mortality. However, these findings should be interpreted with caution due to the substantial clinical heterogeneity and the predominance of observational single-arm studies among the included literature.

## 1 Introduction

Intracranial aneurysms (IAs) are focal pathological dilatations of cerebral arteries, with a global prevalence of approximately 3.2% (1). Most IAs are asymptomatic and are often detected incidentally during imaging for other neurological conditions (2). The surgical management for unruptured IAs involves placing a metal clip on the aneurysm neck, but the procedure is highly invasive and not always possible (3–5). Endovascular management involves the placement of coils within the aneurysm to induce clot formation and occlude the aneurysmal sac (3–5). Still, there are possible complications like thromboembolism and IA rupture. In contrast, ruptured IAs can lead to aneurysmal subarachnoid hemorrhage, a life-threatening event with a 24-hour mortality of up to 25% and a 3-month mortality approaching 50% (6, 7). Despite treatment, ruptured IAs are associated with a 40% mortality rate (1), and survivors might suffer from severe functional impairments (8).

Flow diverters (FDs) are stents placed in the parent artery at the level of the IAs to disrupt the intra-aneurysmal blood flow, decreasing the transmural pressure gradient and the risks of rupture (9). FDs have become feasible and effective for treating most IAs, and their indications are constantly extended, including distal aneurysm locations. FDs are increasingly used in the endovascular management of both unruptured and ruptured IAs, with expanding evidence supporting their efficacy, safety, and unique clinical indications (10–12). Their role continues to evolve as device technology improves and clinical experience grows. Although there have been numerous studies on applying FDs in IAs, most are small-sample-size studies. Two meta-analyses were performed to provide stronger evidence on the effectiveness and safety of FDs for IA, but each focused on a single FD brand, such as Pipeline Flex™ [18] or Silk™ [19], whereas the meta-analysis by Fiorella et al. (13) focused exclusively on unruptured small/medium IAs of the internal carotid artery. Hence, there is a need to examine FDs for IAs more comprehensively, taking into consideration additional studies and trials published since those meta-analyses were performed.

Therefore, this meta-analysis aimed to examine the efficacy and safety of FDs for the treatment of IAs, without restriction to aneurysm rupture status. The results could provide additional high-level evidence for managing IAs using FDs.

## 2 Methods

This meta-analysis was reported according to the Preferred Reporting Items for Systematic Reviews and Meta-Analyses (PRISMA) guidelines and checklist (14, 15). The review was conducted in accordance with the prespecified internal protocol, and no substantive deviations were made during the conduct of the study.

### 2.1 Search strategy

The PubMed, EMBASE, and Cochrane databases were searched for eligible papers published from database inception up to April 11, 2023. The search strategy was formulated using the terms “Intracranial aneurysm” and “Flow-diverting devices”. The detailed search strategies are shown in Supplementary Table S1. All references from the retrieved studies were further examined to identify additional relevant studies.

### 2.2 Study selection

This meta-analysis was designed according to the PICO (population, intervention, control, and outcomes) methods (16). The population was those with IA. The intervention was any FD treatment, including p64™, Tubridge™, Surpass™, Pipeline™, Derivo™, Fred™, Silk™ and PED™. The outcomes were procedural success rate, retreatment, postoperative complete/near-complete occlusion rate, and modified Rankin scale (mRS) scores. The safety outcomes were IA rupture, mortality, postoperative neurogenic death, the incidence of ischemic/thromboembolic, hemorrhagic, treatment-related complications, and the incidence of in-stent restenosis (ISR).

The inclusion criteria were (1) patients with IA, irrespective of the IA shapes, locations, sizes, etc., (2) patients receiving FDs treatment, and (3) all types of clinical studies, including clinical trials, prospective observational studies, and retrospective studies. The exclusion criteria were (1) no efficacy results, (2) studies including fewer than 50 patients treated with FDs treatment. The primary rationale for excluding small-sample studies was based on the statistical instability of rare safety outcomes, which are highly susceptible to zero-event data and extreme proportion estimates. (3) studies with a follow-up time of <6 months, (4) studies with coiling therapy combined with FDs or the proportion of patients received coiling therapy combined with FDs was >15%, (5) using intracranial stent Neuroform Atlas™ or WEB™ due to their different mechanism from the other FDs, (6) conference abstracts, reviews, meta-analyses, preclinical studies, commentaries, or letters to the editor, (7) published in languages other than English.

Two independent investigators conducted the screening and inclusion of the studies. A third investigator resolved any disagreement.

### 2.3 Data extraction

The data were extracted from all studies that could be included. Two investigators extracted the data independently. The extracted data were compared, and discrepancies were resolved through discussion. Data extraction was carried out independently of statistical analysis.

The basic information, treatment, efficacy, and safety outcomes were extracted from each paper. The basic information included the first author, years (publication and start of the study), study type (prospective/retrospective, observational/clinical trial), number of patients, age, sex, number, location, shape, and size of the IAs, and all data pertaining to the outcomes. Outcomes (e.g., aneurysm occlusion, mRS, ISR, neurologic death, and procedure-related complications) were defined according to the original reports; when available, definitions were extracted verbatim, although most studies did not provide explicit definitions.

Occlusion outcomes were extracted as reported in the original studies. Complete occlusion was defined according to each study's original grading system, corresponding to the highest angiographic grade [e.g., Raymond-Roy Occlusion Classification Scale (RROCS) grade I, O'Kelly–Marotta (OKM) grade D, Szikora grade I, modified Cekirge–Saatci grades I, and Montreal grade III]. Complete or near-complete occlusion was grouped as adequate occlusion for pooled analysis (e.g., RROCS I–II, OKM C–D, Szikora grades I–II, modified Cekirge–Saatci grades I–II, and Montreal grades I–III). The generally accepted angiographic definition of ISR is >50% stenosis within the stent. However, most primary studies did not report this definition; therefore, we extracted ISR rates as reported in each original study without retrospectively applying the universal criterion. Because excluding studies without a clear definition would have led to a substantial reduction in the sample size, no definition based sensitivity analysis was performed. Imaging modalities included digital subtraction angiography (DSA), computed tomography angiography (CTA), magnetic resonance angiography (MRA), magnetic resonance imaging (MRI), and computed tomography (CT) as reported in the original studies, and were not uniformly standardized across studies.

### 2.4 Quality assessment

The risk of bias in randomized controlled trials (RCTs) was assessed using the Cochrane Risk of Bias Assessment (ROB2) (17). The risk of bias for single-arm clinical trials was assessed using the MINORS scale (18). The risk of bias in observational studies was assessed using the Newcastle-Ottawa Scale (NOS) (19). The studies were independently evaluated for quality by two investigators. Discrepancies were solved by discussion with a third investigator.

### 2.5 Statistical analysis

All statistical analyses were conducted using R 4.2.2. The outcome measures in this study were all categorical variables; the original data from the included studies were extracted and merged to calculate the total incidence and 95% confidence intervals (CIs ) (20). Cochran's *Q*-test and *I*^2^ were used to assess statistical heterogeneity among studies; *I*^2^ > 50% and a *Q*-test *P* < 0.10 indicate high heterogeneity (21). When heterogeneity among studies was high, the random-effects model was used; otherwise, the fixed-effects model was used (20). Two-sided *P*-values <0.05 were considered statistically significant. Subgroup analyses were conducted by device brand. The potential publication bias was assessed using Egger's test.

## 3 Results

### 3.1 Literature screening and inclusion

**Figure 1:**
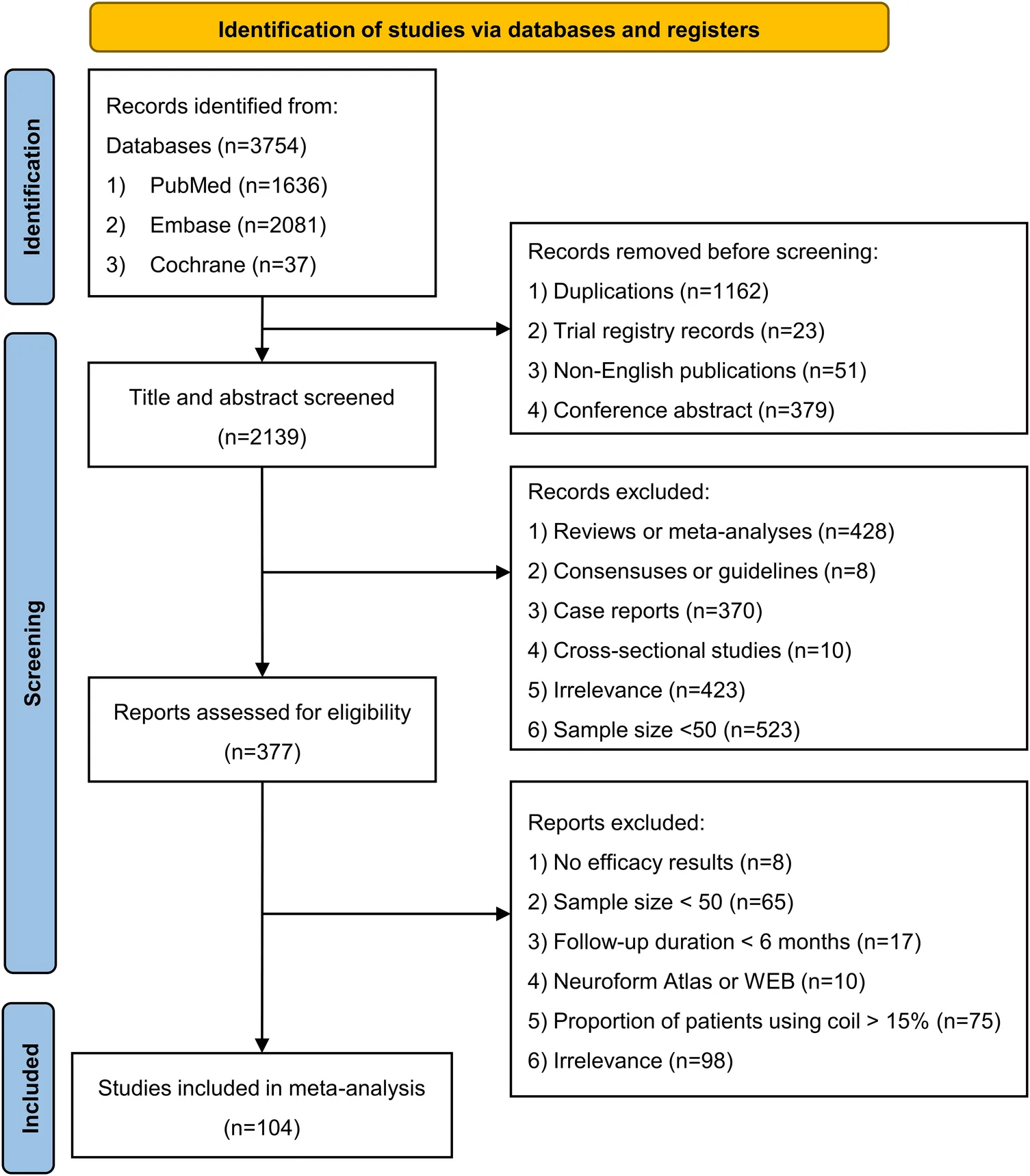
PRISMA flowchart.

The initial search from the three databases yielded 3,754 records (1,636 from PubMed, 2,081 from EMBASE, and 37 from Cochrane). Duplications, trial registry records, non-English papers, and conference abstracts were excluded, leaving 2,139 records. During title and abstract screening, studies with an improper design for the present meta-analysis or with a sample size <50 were excluded, leaving 377 records for full-text review. At the full-text review stage, studies were further assessed for eligibility, and 104 studies were finally included in this meta-analysis. Exclusions at this stage were due to predefined eligibility criteria, including efficacy results, sample size, follow-up duration, and device-related factors (Figure 1).

The characteristics of the 104 studies included are shown in Supplementary Table S2. There were eight single-arm clinical trials, two RCTs, and 94 observational studies. The sample size ranged from 50 to 1,171 patients, totaling 18,990 patients.

### 3.2 Literature quality assessment

According to the quality assessment criteria, in the two RCTs included in the meta-analysis, the risk of bias arising from the randomization process for Raymond et al. (22) was high, and the overall evaluation was considered high risk. The risk of bias for Liu et al. (23) was assessed as an unknown risk. The quality of the observational studies and single-arm clinical trials is shown in Supplementary Tables S3 and S4, respectively. Quality assessment using the NOS for 94 observational studies revealed that 14 studies scored 4 points, 35 studies scored 5 points, 8 studies achieved 6 points, 31 studies achieved 7 points, and 6 studies attained 8 points (Supplementary Table S3). The MINORS scores of the 8 single-arm clinical trials were predominantly distributed between 8 and 12 out of 16 (Supplementary Table S4). All studies consistently lost points in item 5 (unbiased assessment of the study endpoint), indicating a common limitation in outcome assessment.

### 3.3 Effectiveness

**Figure 2:**
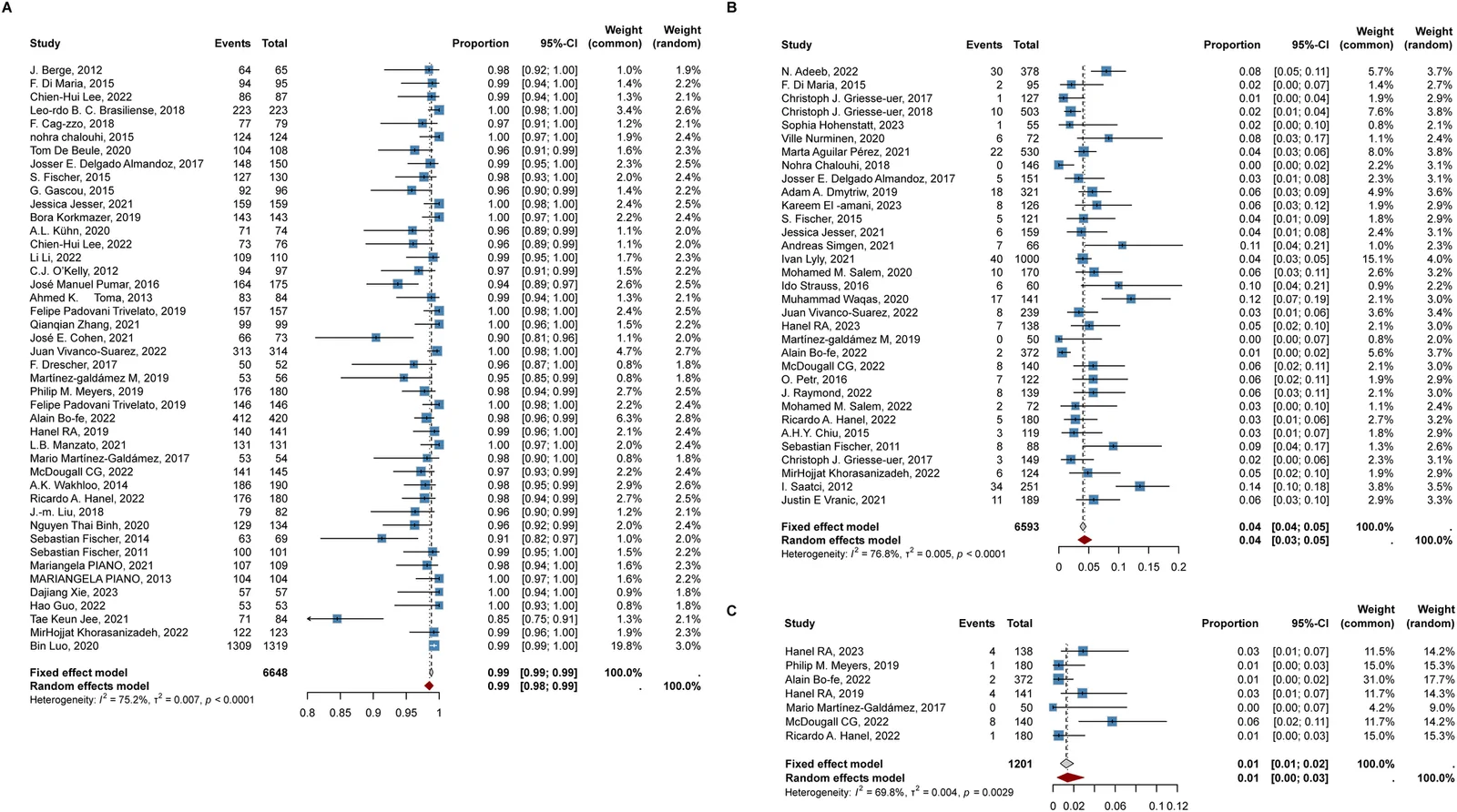
Forest plot of **(A)** procedural success rate, **(B)** total postoperative retreatment rate and **(C)** retreatment rate in the first year after the procedure.

The procedural success rate was 99% (95% CI: 98%–99%; 44 studies) (Figure 2A). Thirty-three studies reported the proportion of patients who received retreatment, yielding a pooled rate of 4% (95% CI: 3%–5%) (Figure 2B). During the first year after treatment, the proportion of patients who received retreatment was 1% (95% CI: 0%–3%; seven studies) (Figure 2C).

**Figure 3:**
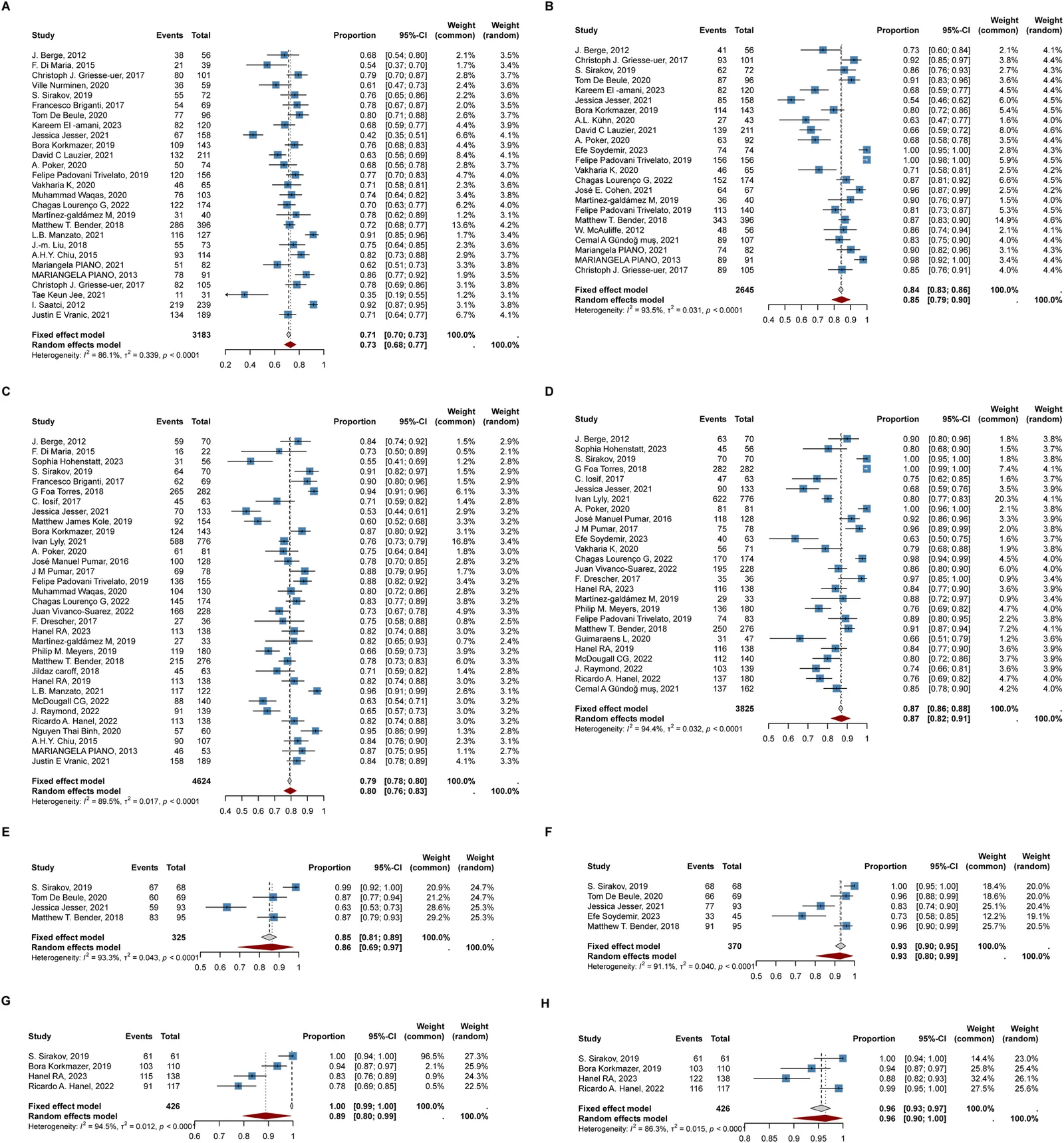
Forest plot of **(A)** complete occlusion rate at 6 months after the procedure, **(B)** complete/near complete occlusion rate at 6 months after the procedure, **(C)** complete occlusion rate at 12 months after the procedure, **(D)** complete/near complete occlusion rate at 12 months after the procedure, **(E)** complete occlusion rate at 24 months after the procedure, **(F)** complete/near complete occlusion rate at 24 months after the procedure, **(G)** complete occlusion rate at 36 months after the procedure and **(H)** complete/near complete occlusion rate at 36 months after the procedure.

The pooled complete occlusion and complete/near complete occlusion rates were at 73% (95% CI: 68%–77%; 27 studies) (Figure 3A) and 85% (95% CI: 79%–90%; 23 studies) (Figure 3B) at 6 months, respectively; 80% (95% CI: 76%–83%; 33 studies) (Figure 3C) and 87% (95% CI: 82%–91%; 26 studies) (Figure 3D) at 12 months, respectively. The pooled complete occlusion and complete/near complete occlusion rates were 86% (95% CI: 69%–97%; four studies) (Figure 3E) and 93% (95% CI: 80%–99%; five studies) at 24 months (Figure 3F), respectively, and 89% (95% CI: 80%–99%; four studies) (Figure 3G) and 96% (95% CI: 90%–100%; four studies) (Figure 3H), respectively, at 36 months.

**Figure 4:**
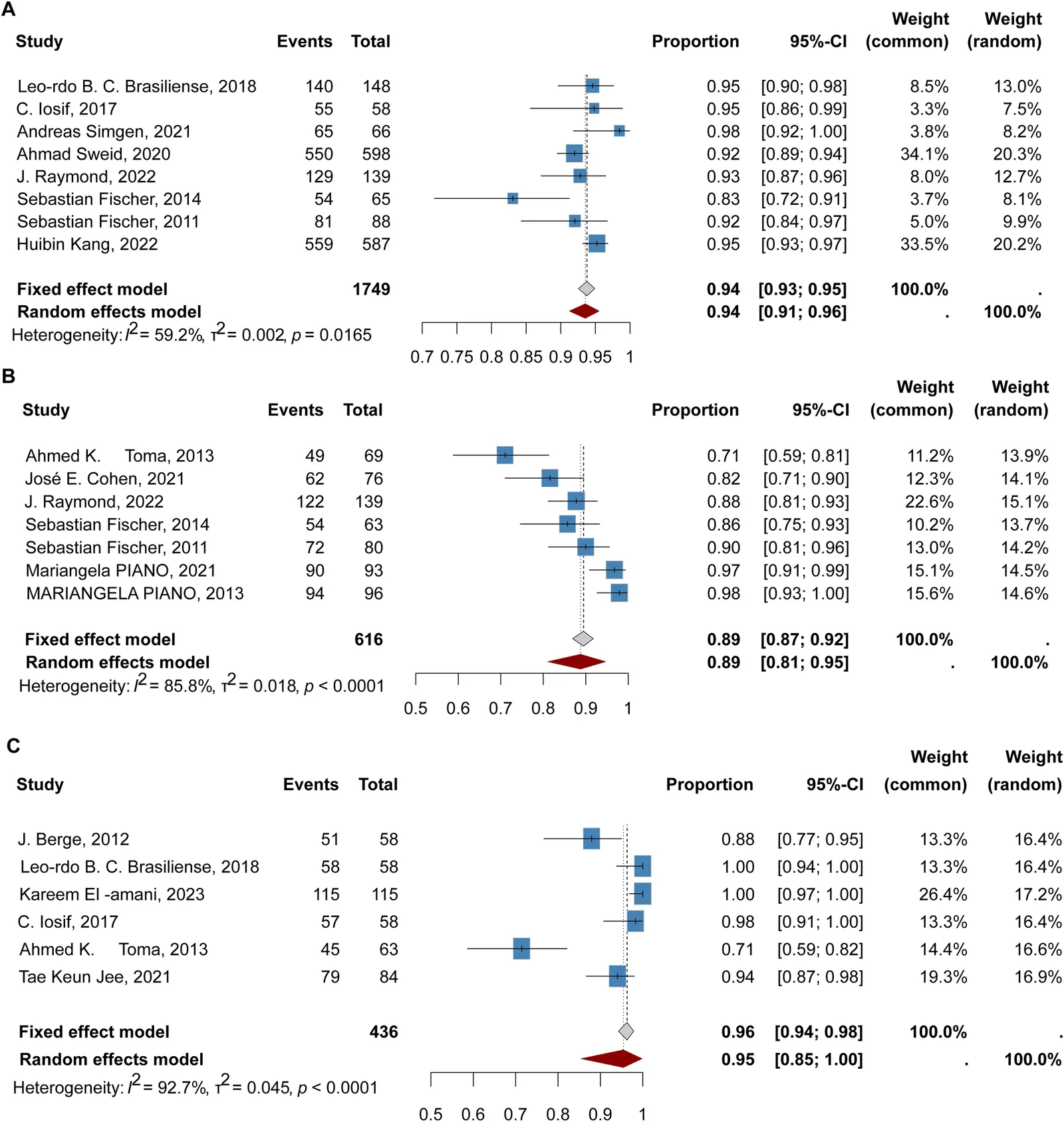
Forest plot of **(A)** proportion of patients with a modified Rankin scale (mRS) score ≤2 in the first month after the procedure, **(B)** proportion of patients with mRS score ≤2 in the third month after the procedure and **(C)** proportion of patients with mRS score ≤2 in the 6th month after the procedure.

The proportions of patients with mRS score ≤2 at 1, 3, and 6 months after the procedure were 94% (95% CI: 91%–96%; eight studies) (Figure 4A), 89% (95% CI: 81%–95%; eight studies) (Figure 4B), and 95% (95% CI: 85%–100%; six studies) (Figure 4C), respectively.

### 3.4 Safety

**Figure 5:**
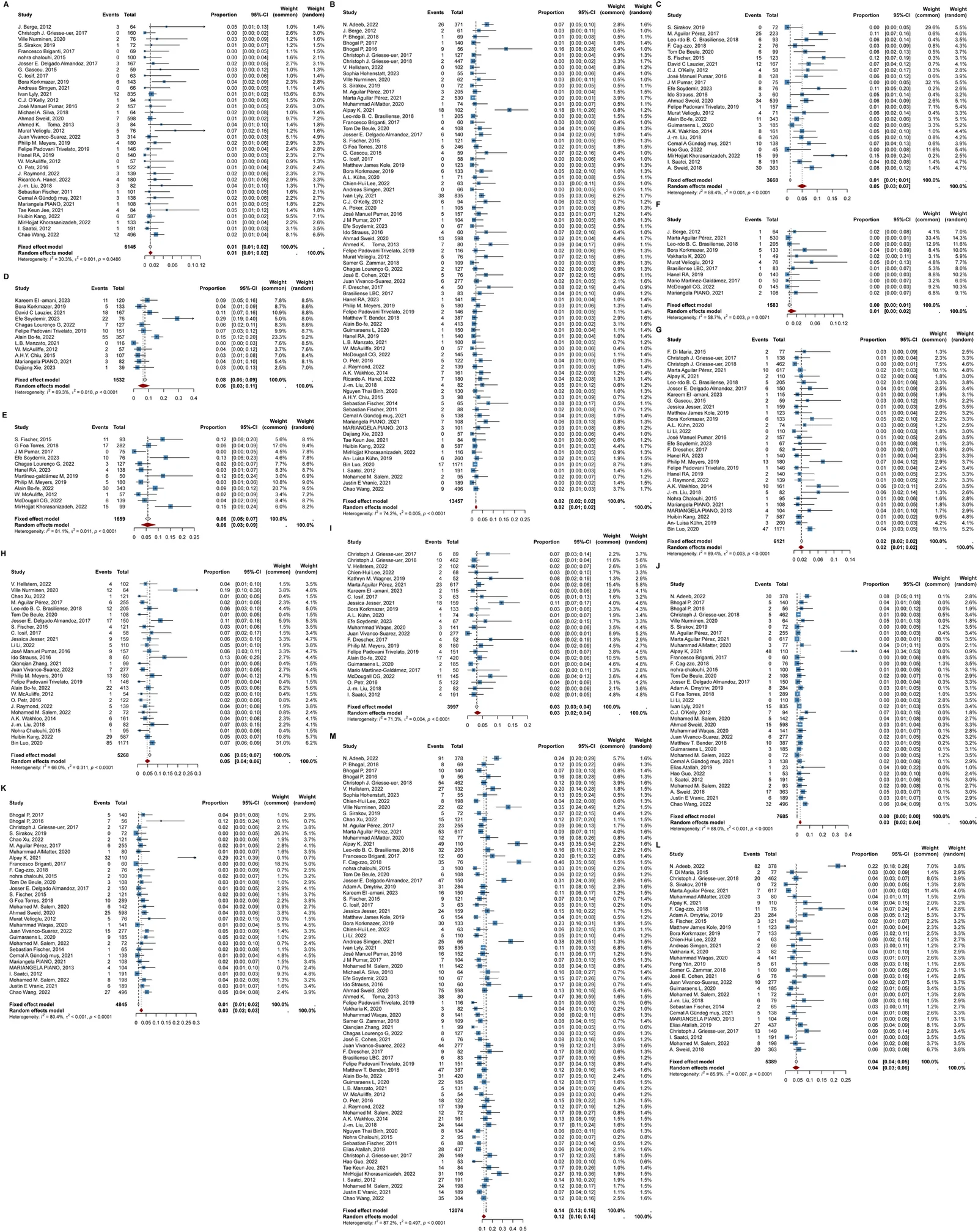
Forest plot of the **(A)** aneurysm rupture rate, **(B)** all-cause mortality rate, **(C)** incidence of total in-stent restenosis (ISR), **(D)** incidence of ISR at 6 months after the procedure, **(E)** incidence of ISR at 12 months after the procedure, **(F)** neurogenic death rate at 1 month after the procedure, **(G)** hemorrhagic rate at 1 month after the procedure, **(H)** ischemic rate at 1 month after the procedure, **(I)** thromboembolic rate at 1 month after the procedure, **(J)** hemorrhagic rate with unspecified follow-up duration, **(K)** ischemic rate with unspecified follow-up duration, **(L)** thromboembolic rate with unspecified follow-up duration, and **(M)** incidence of treatment-related complications.

The aneurysm rupture and all-cause mortality rates were 1% (95% CI: 1%–2%; 35 studies) (Figure 5A) and 2% (95% CI: 1%–2%; 77 studies) (Figure 5B), respectively. The overall incidence of ISR was 5% (95% CI: 3%–7%; 24 studies) (Figure 5C), and almost the same rates were observed at 6 (6%, 95% CI: 3%–11%; 12 studies) and 12 (6%, 95% CI: 3%–9%; 12 studies) months (Figures 5D,E).

At 1 month after the procedure, neurogenic death was reported in 1% (95% CI: 0%–2%; 11 studies) of the patients (Figure 5F), hemorrhage in 2% (95% CI: 1%–2%; 30 studies) (Figure 5G), ischemia in 5% (95% CI: 4%–6%; 27 studies) (Figure 5H) and thromboembolism in 3% (95% CI: 2%–4%) (Figure 5I). For the studies that reported the complication rates with unspecified follow-up duration, hemorrhage was observed in 3% (95% CI: 2%–4%; 35 studies) of the patients (Figure 5J), ischemia in 3% (95% CI: 2%–3%; 30 studies) (Figure 5K) and thromboembolism in 4% (95% CI: 3%–6%; 31 studies) (Figure 5L). The incidence of treatment-related complications was 12% (95% CI: 10%–14%) (Figure 5M).

### 3.5 Subgroup analysis by brand

**Figure 6:**
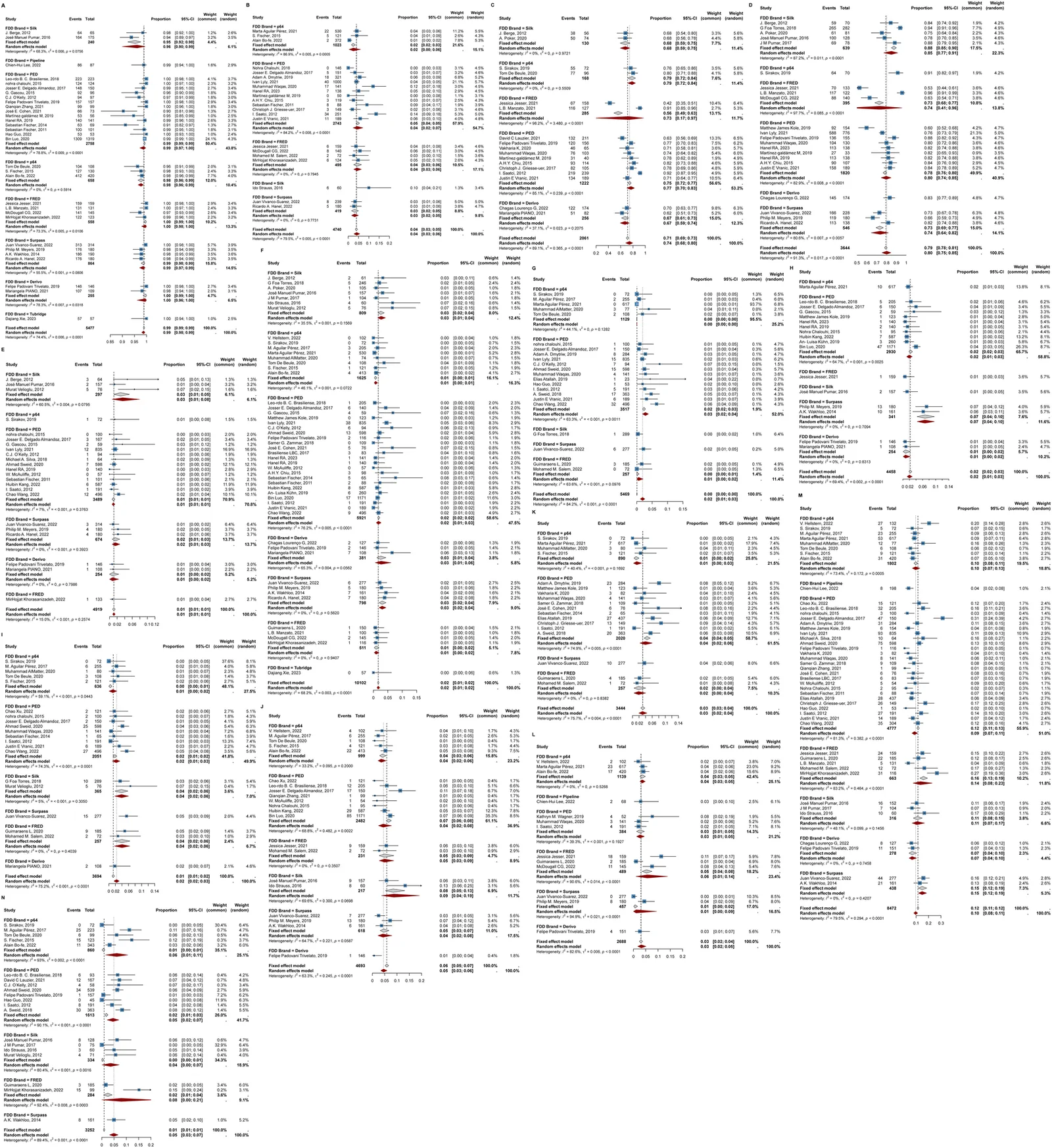
Subgroup analysis by brand. **(A)** Procedural success rate, **(B)** total postoperative retreatment rate, **(C)** complete occlusion rate at 6 months after the procedure, **(D)** complete occlusion rate at 12 months after the procedure, **(E)** aneurysm rupture rate, **(F)** all-cause mortality rate, **(G)** hemorrhagic rate with unspecified follow-up duration, **(H)** hemorrhagic rate at 1 month after the procedure, **(I)** ischemic rate with unspecified follow-up duration, **(J)** ischemic rate at 1 month after the procedure, **(K)** thromboembolic rate with unspecified follow-up duration, **(L)** thromboembolic rate at 1 month after the procedure, **(M)** incidence of treatment-related complications, and **(N)** incidence of in-stent restenosis (ISR).

The procedural success rate was similar across brands, ranging from 96% to 100% (Figure 6A). For the efficacy endpoints, retreatment rate estimates ranged from 1% to 10% across brands, with broadly overlapping 95% CIs (Figure 6B). At 6 months post-procedure, complete occlusion rates ranged from 67% to 79% (Figure 6C), and at 12 months from 74% to 91% (Figure 6D). In all cases, the associated CIs showed substantial overlap, consistent with sampling variability and precluding any inference of differential device performance.

Rates of aneurysm rupture and all-cause mortality were similar across brands, ranging from 1% to 3% (Figures 6E,F). The incidence of hemorrhagic events after the procedure (unspecified follow-up duration) ranged from 0% to 3% (Figure 6G), and at 1 month post-procedure from 1% to 2% (Figure 6H). Ischemic events after the procedure (unspecified follow-up) ranged from 1% to 5% (Figure 6I), and at 1 month from 4% to 9% (Figure 6J). Thromboembolic events after the procedure (unspecified follow-up) ranged from 1% to 4% (Figure 6K), and at 1 month from 1% to 6% (Figure 6L). Treatment-related complications ranged from 4% to 15% (Figure 6M), and ISR from 4% to 8% (Figure 6N). Across all safety endpoints, the 95% CIs overlapped broadly, and no brand consistently demonstrated a distinct safety profile.

### 3.6 Publication bias

The results of publication bias are shown in Supplementary Table S5. Postoperative occlusion rate at 36 months after the procedure, incidences of hemorrhagic/ischemic events after the procedure (unspecified follow-up duration), ischemic event/neurogenic death at 1 month after the procedure, hemorrhagic event at 12 months after the procedure, overall incidence of ISR, and treatment-related complications might have publication bias (all *P* < 0.05). The corresponding funnel plots for all outcomes are presented in Supplementary Figures S1 and S2. Overall, visual inspection of the funnel plots was generally consistent with the results of Egger's test.

## 4 Discussion

This meta-analysis summarized multiple studies on the treatment of IA with FDs. The procedural success rate was 99%, and the postoperative retreatment rate was only 4%. The pooled complete occlusion and complete/near-complete occlusion rates at 6 months after FDs reached 73% and 85%, respectively, and subsequently both exceeded 90% at 12, 24, and 36 months after the procedure. Within 1 year after FDs implantation, the proportion of patients with an mRS score ≤2 was generally >90%, and the aneurysm rupture and all-cause mortality rates were relatively low (1% and 2%). Regarding safety, the incidence of postoperative cerebrovascular events was below 5%, and the incidence of treatment-related complications was 12%. Subgroup analyses by FD brand suggested that, numerically, p64™ had a higher occlusion rate. Silk™ had a relatively higher incidence of ischemic events, whereas Surpass™ demonstrated a numerically higher rate of hemorrhagic events but a low rate of thromboembolic events.

Several meta-analyses examined the efficacy of FDs for IAs. For instance, Lv et al. (24) included 29 studies and 1,524 patients and reported an overall technical failure and complication rate of 9.3%. Still, they could not examine efficacy and safety in detail. Fiorella et al. (13) reported a 12-month complete occlusion rate of 75% but exclusively in patients with internal carotid artery IAs. Zhou et al. (25) observed an overall occlusion rate of 83% and a technical success rate of 97% in patients with IA treated with FDs. Cagnazzo et al. (26) showed a complete or near-complete occlusion rate of 79% in patients with middle cerebral artery IAs. The present study of 104 studies and 18,990 patients could examine the complete occlusion and complete/near complete occlusion rates at multiple time points, including 6 (73% and 85%), 12 (80% and 87%), 24 (86% and 93%), and 36 (89% and 96%) months after the procedure. Recent studies have reported numerically similar occlusion outcomes to those observed in the present analysis (27–30). However, interpretation of these results should consider substantial heterogeneity across studies. Imaging modalities varied widely, including DSA, MRA, and CTA, and different modalities were often used at different follow-up time points. Given their different sensitivities for detecting residual flow, this may introduce variability in reported occlusion rates (31, 32). Moreover, the occlusion grading systems employed were heterogeneous, including Raymond-Roy, O'Kelly-Marotta, modified Cekirge–Saatci, and the Montreal grading system. Although these systems differ in their definitions and granularity of “complete” and “near-complete” occlusion (33–36), such pooling represents an approximate methodological approach that may introduce some degree of heterogeneity or misclassification bias. Nevertheless, it is a widely accepted and commonly used practice in this field, which improves cross-study comparability while maintaining methodological rigor (37, 38). Taken together, heterogeneity in imaging modalities and grading systems represents an important factor to consider when interpreting occlusion outcomes in this meta-analysis.

The procedural success rate was 99%, higher than in previous meta-analyses, but the present study included more recent studies, and the higher success rate could be due to better operative experience. In addition, the present meta-analysis examined neurological function and found that 89%–96% of patients had an mRS score of ≤2 within 6 months after the procedure, suggesting a favorable prognosis (39). The reported 94% at month 1 and the numerically lower rate of 89% at month 3 might be attributed to the different studies included in the 1- and 3-month analyses, which limited the longitudinal comparison.

In the present meta-analysis, the rates of IA rupture, mortality, ISR, and 1-month neurogenic death, hemorrhage, ischemia, and thromboembolism were low (all ≤6%). The present meta-analysis allowed analysis of a larger number of complications, in greater detail than previous meta-analyses. Indeed, Lv et al. (24) (29 studies and 1,524 patients) reported a higher complication rate (14%) than in the present study, while morbidity and mortality rates were 6.6%. They identified that fusiform, dissecting, and circumferential IAs, the posterior circulation, and peripheral location were associated with higher complication rates. Fiorella et al. (13) (internal carotid artery IAs; 41 studies and 2,614 patients) reported a primary complication rate of 8%. Cagnazzo et al. (26) (middle cerebral artery IAs; 12 studies and 244 patients) reported a rupture rate of 0.4% per aneurysm-year, a rate of FDs-related complications of 21%, and a mortality rate of 2%. The disparities between different meta-analyses, including the present study, might be attributed to differences in population and reporting bias among the original included studies. Notably, most included studies lacked standardized reporting of antiplatelet regimens, preventing analysis of complication rates across different antiplatelet strategies. Given that hemorrhagic, ischemic, and thromboembolic events are strongly influenced by the antiplatelet strategies employed (40, 41), these inconsistencies should be carefully considered when interpreting the pooled complication rates.

Owing to the large number of studies and patients included, the present meta-analysis could perform subgroup analyses by FDs brand. In previous meta-analyses, the 30-day mortality rate for the Pipeline™ FDs was 0.8%, the incidence of treatment-related complications was 10.1%, and the procedural success rate was 99.3% [18], results similar to those of the present study. Using the Surpass™ FDs leads to an occlusion rate of 73%, rates of thromboembolic and hemorrhagic complications of 7% and 3%, and a mortality rate of 5% (42). In addition, in previous meta-analyses, the complete occlusion rate of the Silk™ FDs was around 81% (43, 44), and the mortality rate was 2.84% (43). When examined by brand, numerical variation was apparent in the occlusion rates and complication profiles observed across the aggregated device subgroups. However, these observations must be interpreted with caution. The results are derived from pooled estimates of single-arm studies, with differences in study populations, aneurysm characteristics, follow-up durations, and reporting time points across the contributing studies. Critically, in the absence of head-to-head studies, no causal attribution can be made regarding the observed numerical differences. The wide and substantially overlapping confidence intervals around the brand-specific estimates further indicate that the numerical spread is compatible with sampling variability rather than true performance divergence. Therefore, the device-level findings of this analysis should be considered strictly exploratory and hypothesis-generating. They do not provide evidence for the superiority or inferiority of any particular flow diverter brand.

This meta-analysis has several limitations. First, most included studies were retrospective single-arm observational studies, which are inherently subject to selection bias and confounding, limiting causal inference. Second, substantial clinical and methodological heterogeneity existed across studies, including differences in aneurysm characteristics, rupture status, device selection, antiplatelet regimens, and operator experience, imaging modalities (DSA, CTA, MRA) and occlusion grading systems across studies. In addition, key outcomes such as in-stent restenosis and neurologic death were not explicitly defined across studies, introducing potential misclassification bias. Third, these issues, together with insufficient reporting, and the narrow age distribution of the study populations (predominantly 45–65 years), precluded meaningful subgroup analyses, including the assessment of the potential modifying effect of age, and limited device-specific interpretation due to the lack of head-to-head comparisons between different FDs. In addition, outcomes reported at later follow-up points likely represent cumulative incidence rather than events at specific time points, introducing temporal heterogeneity and limiting the distinction between early and late events. Fourth, exclusion of small-sample studies (<50 patients) may have introduced selection bias toward higher-volume centers or more experienced operators; however, such studies are prone to sparse-event data, zero-event inflation, and increased heterogeneity, which may affect the stability of pooled estimates in rare-event settings. Finally, the literature search was conducted up to April 2023, and more recent studies were not included. Publication bias and potential patient overlap in registry-based studies may also have influenced the pooled estimates. These limitations underscore the need for prospective, high-quality studies with standardized outcome definitions and reporting in this field.

In conclusion, this meta-analysis suggests that FDs achieve high occlusion and procedural success rates for IA, resulting in a favorable neurological prognosis. The incidence of postoperative cerebrovascular events and treatment-related complications is low. Exploratory subgroup analyses showed numerical differences among different brands of FDs. P64™ showed numerically higher occlusion rates and lower hemorrhagic, ischemic, and thromboembolic event rates, while Surpass™ showed higher hemorrhagic but lower thromboembolic event rates. However, given the predominance of non-comparative study designs, limited adjustment for confounding factors, and insufficient stratified reporting across studies, these pooled estimates should be interpreted as overall summary measures rather than condition-specific or causal effects. High-quality clinical research in the future is still needed to provide data to support comparisons between different brands of FDs. More recent studies and newly developed FDs published after the search period may further refine comparative outcomes and should be considered in future updated meta-analyses.

## Data Availability

The original contributions presented in the study are included in the article/Supplementary Material, further inquiries can be directed to the corresponding author.
