# Supplementary material for: Efficacy and safety of flow diverters for the treatment of intracranial aneurysms: a systematic review and meta-analysis
Source: Front Surg. 2026 Aug 10;13:1788189. doi: 10.3389/fsurg.2026.1788189 (PMC13500760; doi:10.3389/fsurg.2026.1788189)
Supplement: Supplementary file 1 [file Supplementaryfile1.docx]

**Supplementary materials**

**Supplementary Table S1.** Search strategies

| **PubMed** | | |  | | | **Search strategy** | | | **Numbers** | | |
| --- | --- | --- | --- | --- | --- | --- | --- | --- | --- | --- | --- |
| **Patient** | | | **#1** | | | ((Intracranial Aneurysm[MeSH Terms]) OR (cerebral aneurysm*)) OR (brain aneurysm*) | | | 48,139 | | |
| **Intervention** | | | **#2** | | | (((((((flow divert*) OR (p64)) OR (Tubridge)) OR (Surpass)) OR (Derivo)) OR (SILK AND (Balt Extrusion))) OR ((Pipeline OR (Pipeline Flex) OR PED) AND (Medtronic))) OR ((FRED AND MicroVention) OR (Flow Re-Direction Endoluminal Device System)) | | | 20,703 | | |
|  | | | **#3** | | | #1 AND #2 | | | 1636 | | |
| Embase | |  | | | Search strategy | | | Numbers | | |  |
| Patient | | **#1** | | | 'intracranial aneurysm'/exp OR 'aneurysm, intracranial' OR 'cerebral aneurysm' OR 'brain aneurysm' | | | 42,334 | | |  |
| Intervention | | #2 | | | 'flow divert*' OR p64 OR tubridge OR surpass OR derivo OR 'flow-redirection endoluminal device system' OR (silk AND balt) OR (fred AND microvention) OR (medtronic AND (pipeline OR (pipeline AND flex) OR ped)) | | | 9764 | | |  |
|  | | **#3** | | | #1 AND #2 | | | 2081 | | |  |
| Cochrane |  | | |  | | |  | | |  |  |
| Patient | #1 | | | MeSH descriptor: [Intracranial Aneurysm] explode all trees | | | 599 | | |  |  |
|  | #2 | | | (cerebral aneurysm):ti,ab,kw OR (brain aneurysm):ti,ab,kw | | | 1529 | | |  |  |
|  | #3 | | | #1 OR #2 | | | 1728 | | |  |  |
| Intervention | #4 | | | (((((((flow divert*) OR (p64)) OR (Tubridge)) OR (Surpass)) OR (Derivo)) OR (SILK AND Balt)) OR ((Pipeline OR (Pipeline Flex) OR PED) AND (Medtronic))) OR ((FRED AND MicroVention) OR (Flow Re-Direction Endoluminal Device System)) | | | 1020 | | |  |  |
|  | #5 | | | #3 AND #4 | | | 37 | | |  |  |

**Supplementary Table S2.** Characteristics of included studies

| No | First author | Year | Starting and ending time | Study type | Total sample size | Average age | Median age | Whether to combine coiling therapy | Number of FDDs | Female percentage | Number of aneurysms | FDD brand |
| --- | --- | --- | --- | --- | --- | --- | --- | --- | --- | --- | --- | --- |
| 1 | Adeeb (1) | 2022 | 2011.1~2020.1 | Retrospective study | 378 | - | 57 | Yes | - | 50.00% | 378 | PED; FRED |
| 2 | Berge (2) | 2012 | 2008.9~2009.12 | Retrospective study | 65 | 54 | - | Yes | 73 | 78.46% | 77 | Silk |
| 3 | Bhogal (3) | 2019 | 2009.2~2018.2 | Retrospective study | 69 | 55 | - | - | 69 | 89.86% | 169 | PED; p64; Surpass |
| 4 | Bhogal (3) | 2017 | 2009.9~2016.7 | Retrospective study | 140 | 56.2 | - | - | 140 | 77.86% | 147 | FDS; PED |
| 5 | Bhogal (4) | 2017 | 2009.2~2016.4 | Retrospective study | 56 | 63.5 | - | Yes | 338 | 19.64% | 58 | PED; p64 |
| 6 | F. Di Maria (5) | 2015 | 2006.2~2013.12 | Retrospective study | 138 | 49.7 | - | Yes | 77 | 80.43% | 162 | PED; Silk; Surpass |
| 7 | Griessenauer (6) | 2017 | 2010.9~2015.10 | Retrospective study | 127 | 54 | - | - | - | 87.40% | 160 | PED; Silk |
| 8 | Griessenauer (7) | 2018 | 2012~2017 | Retrospective study | 447 | 55.2 | - | Yes | 462 | 80.98% | 503 | PED; FRED |
| 9 | V. Hellstern (8) | 2022 | 2020.3~2021.12 | Retrospective study | 102 | 58.1 | - | - | 147 | 75.49% | 132 | p64 |
| 10 | Hohenstatt (9) | 2023 | 2009~2021 | Retrospective study | 55 | 62 | - | - | - | 65.45% | 56 | PED; PED Shield, FRED; Silk; p48 |
| 11 | Lee (10) | 2022 | 2018.10~2020.7 | Retrospective study | 70 | 57 | - | - | 70 | 77.14% | 87 | Pipeline flex; flow redirection endoluminal device |
| 12 | Nurminen (11) | 2020 | 2014.2~2019.1 | Retrospective study | 62 | - | 54 | Yes | 72 | 83.87% | 76 | PED; Surpass; Fred; Derivo |
| 13 | Sirakov (12) | 2019 | 2015.3~2018.11 | Retrospective study | 72 | 54.8 | - | - | 72 | 55.56% | 72 | p64 |
| 14 | Wagner (13) | 2019 | 2012.8~2018.6 | Retrospective study | 52 | 59 | - | - | - | 75.00% | 52 | PED |
| 15 | Xu (14) | 2022 | 2014.11~2019.10 | Retrospective study | 189 | 53 | - | Yes | 195 | 79.89% | 194 | PED |
| 16 | Aguilar Pérez (15) | 2017 | 2011.12.14~2015.12.31 | Retrospective study | 205 | - | 57 | - | 252 | 71.71% | 223 | p64 |
| 17 | Aguilar Pérez (16) | 2021 | 2011.12~2019.12 | Retrospective study | 530 | - | 55.9 | - | 679 | 73.21% | 617 | p64 |
| 18 | AlMatter (17) | 2020 | 2016.11~2019.9 | Retrospective study | 74 | 57 | - | - | - | 83.78% | 77 | p64 |
| 19 | Alpay (18) | 2021 | 2012.1~2019.12 | Retrospective study | 110 | 55.7 | - | Yes | 128 | 58.18% | 110 | PED; PED shield; Surpass Stream Lines; FRED; FRED JR; Derivos FD; Surpass Evolves; Silk FD |
| 20 | Brasiliense (19) | 2018 | 2014.7~2016.3 | Retrospective study | 205 | 55.7 | - | Yes | 223 | 80.49% | 223 | PED Flex |
| 21 | Briganti (20) | 2017 | 2008.11~2015.11 | Retrospective study | 60 | 57 | - | Yes | 63 | 80.00% | 69 | PED; Silk; FRED; p64 |
| 22 | Cagnazzo (21) | 2018 | 2011.1~2018.1 | Retrospective study | 76 | 54.5 | - | Yes | - | 67.11% | 76 | LEO stent |
| 23 | Chalouhi (22) | 2018 | 2011~2015 | Retrospective study | 146 | 55.2 | - | Yes | - | 88.36% | 146 | PED |
| 24 | Chalouhi (23) | 2015 | 2011.5~2013.9 | Retrospective study | 100 | - | - | Yes | 124 | 89.00% | 100 | PED |
| 25 | De Beule (24) | 2020 | 2014.3~2019.7 | Retrospective study | 108 | 57 | 59 | Yes | - | 80.56% | 109 | p64 |
| 26 | Delgado Almandoz (25) | 2017 | 2011.3.10~2015.11.5 | Retrospective study | 140 | 55.4 | 56 | Yes | - | 77.86% | 167 | PED |
| 27 | Dmytriw (26) | 2019 | 2011~2016.3 | Retrospective study | 284 | - | 57 | Yes | - | 88.38% | 321 | PED |
| 28 | El-Naamani (27) | 2023 | 2018~2021 | Retrospective study | 150 | PED group: 53.8;  FRED group: 60.9 | - | No | - | 85.33% | 150 | PED; FRED |
| 29 | Fischer (28) | 2015 | 2012.4~2014.10 | Retrospective study | 121 | - | 58 | Yes | 143 | / | 130 | p64 |
| 30 | Foa Torres (29) | 2018 | 2009.1~2017.1 | Retrospective study | 246 | 51 | - | Yes | 246 | 86.18% | 293 | Silk |
| 31 | Gascou (30) | 2015 | 2009.7~2012.6 | Retrospective study | 59 | 53.7 | - | Yes | 96 | 77.97% | 66 | PED |
| 32 | Iosif (31) | 2017 | 2010.1~2014.12 | Retrospective study | 58 | 52.4 | - | Yes | - | 70.69% | 63 | PED; FRED; Silk |
| 33 | Jesser (32) | 2021 | 2014-2020 | Retrospective study | 150 | 55 | - | Yes | - | 67.33% | 159 | FRED |
| 34 | Kole (33) | 2019 | 2011-2016 | Retrospective cohort study | 123 | 55 | - | No | - | 87.80% | 154 | PED |
| 35 | Korkmazer (34) | 2019 | 2008-2013 | Retrospective study | 133 | 46.3 | - | Yes | 149 | 84.21% | 143 | Silk; PED; FRED |
| 36 | Kühn (35) | 2020 | 2018-2020 | Retrospective study | 74 | 57.5 | - | Yes | - | 86.49% | 86 | PED; Surpass; FRED |
| 37 | Lauzier (36) | 2021 | 2011-2020 | Retrospective cohort study | 159 | 56 | - | - | 167 | 83.65% | 211 |  |
| 38 | Lee (10) | 2022 | 2018-2021 | Retrospective study | 63 | 59 | - | Yes | - | 77.78% | 126 | PED Flex; FRED |
| 39 | Li (37) | 2022 | 2014-2019 | Retrospective study | 110 | 53.7 | - | Yes | 121 | 30.00% | 145 | Tubridge; PED |
| 40 | Simgen (38) | 2021 | 2014-2018 | Retrospective study | 66 | - | 55 | No | - | 75.76% | 66 | PED; Derivo; FRED; p64 |
| 41 | Lylyk (39) | 2021 | 2006-2019 | Retrospective study | 835 | 55.9 | - | Yes | 1214 | 80.36% | 1000 | PED, PED Flex, PED Shield |
| 42 | O’Kelly (40) | 2013 | 2008-2010 | Retrospective cohort study | 97 | 56.5 | - | Yes | - | / | 97 | PED |
| 43 | Poker (41) | 2020 | 2010-2019 | Retrospective study | 105 | 51.2 | - | Yes | - | 67.62% | 127 | Silk Plus |
| 44 | Pumar (42) | 2017 | 2008-2013 | Retrospective study | 157 | 56.2 | 56.7 | Yes | 175 | 75.80% | 180 | Silk |
| 45 | Pumar (43) | 2018 | 2008-2013 | Retrospective study | 104 | 55.2 | 57.1 | - | - | 75.00% | 109 | Silk |
| 46 | Salem (44) | 2020 | 2009-2017 | Retrospective study | 165 | - | 57 | Yes | - | 83.64% | 202 | PED; SAC |
| 47 | Silva (45) | 2018 | 2011-2017 | Retrospective study | 115 | 54.5 | - | Yes | - | 90.43% | 125 | PED |
| 48 | Soydemir (46) | 2023 | 2013-2019 | Retrospective study | 67 | 57.8 | 58 | Yes | 79 | 77.61% | 76 | FREDs; FRED Jr.s; PED; Surpass |
| 49 | Strauss (47) | 2016 | 2008-2013 | Retrospective study | 60 | 57.30 | - | Yes | 62 | 75.00% | 67 | Silk |
| 50 | Sweid (48) | 2020 | 2010.1~2019.12 | Retrospective study | 598 | 55.5 | - | Yes | - | 84.28% | 598 | Classical PED; PED Flex |
| 51 | Toma (49) | 2013 | 2008.3.18~2011.11.10 | Retrospective study | 80 | 54.1 | - | Yes | - | 63.10% | 84 | Pipeline; Silk |
| 52 | Trivelato (50) | 2019 | 2011.11~2016.6 | Retrospective study | 116 | 51.9 | 54 | Yes | - | 89.66% | 157 | PED |
| 53 | Vakharia (51) | 2020 | 2014.6~2016.10 | Retrospective study | 82 | Biaxial catheter: 55.8 Triaxial catheter: 56.0 | - | No | - | 81.71% | 89 | PED: biaxial and triaxial catheter |
| 54 | Velioglu (52) | 2012 | 2008.3~2011.6 | Retrospective study | 76 | 47.9 | - | Yes | - | 76.32% | 87 | Silk |
| 55 | Waqas (53) | 2020 | 2013.1~2017.10 | Retrospective study | 119 | Single PED group: 57.7 Multiple PEDs group: 55.9 | - | No | 182 | =- | 141 | PED |
| 56 | Zammar (54) | 2018 | 2011~2015 | Retrospective study | 109 | 54.4 | - | - | - | 83.49% | 109 | PED |
| 57 | Zhang (55) | 2021 | 2018.1~2020.6 | Retrospective study | 125 | PED flex group: 53.3 PED flex+coil group: 53.2 | - | Yes | - | 75.00% | 140 | PED flex |
| 58 | Chagas Lourenço (56) | 2022 | 2016.12~2019.9 | Retrospective study | 127 | 53.3 | - | Yes | 187 | 85.04% | 177 | Derivo |
| 59 | Cohen (57) | 2021 | 2010.7~2018.9 | Retrospective study | 76 | 42.8 | - | No | 76 | 60.53% | 76 | PED |
| 60 | Vivanco-Suarez (58) | 2023 | 2018.1~2021.6 | Retrospective study | 277 | - | 60 | Yes | 334 | 72.92% | 314 | Surpass |
| 61 | Drescher (59) | 2017 | 2013.3~2016.2 | Retrospective study | 50 | 56.00 | - | Yes | - | 80.00% | 52 | FRED (One case with a combined use of Silk and PEDs) |
| 62 | Brasiliense (60) | 2017 | - | Prospective study | 83 | 61.2 | - | Yes | 106 | 90.36% | 117 | PED |
| 63 | Hanel (61) | 2023 | 2014.7~2015.11 | Prospective study | 141 | - | - | Yes (No specific data) | - | =- | - | PED |
| 64 | Martínez-Galdámez (62) | 2019 | 2015.3~2015.10 | Prospective study | 50 | 53.00 | - | No | 56 | 82.00% | 50 | PED |
| 65 | Meyers (63) | 2019 | 2012.10~2015.11 | Prospective study | 180 | 61 | - | No | - | 91.67% | 180 | Surpass (Stryker Neurovascular, Fremont, CA) |
| 66 | Trivelato (64) | 2019 | 2016.12~2018.10 | Prospective study | 146 | 55.3 | - | Yes | 186 | 82.88% | 183 | Derivo Embolization Device |
| 67 | Bender (65) | 2018 | 2011.8~2016.8 | Retrospective study | 387 | 56.10 | - | Yes | - | 96.12% | 445 | PED; SIlK stent |
| 68 | Bonafe (66) | 2022 | 2015.12~2019.1 | Prospective study | 420 | 55 | - | Yes | 445 | 86.19% | 420 | P64 |
| 69 | Caroff (67) | 2019 | 2011.5~2015.5 | Retrospective study | 63 | PED group: 52;  SILK group: 53 | - | No | - | 80.95% | 63 | PED; SIlK stent |
| 70 | Guimaraens (68) | 2020 | 2015.12~2018.7 (Note: The original methodology was written on July 2018, while the results section was written on July 2019) | Prospective study | 150 | 54.30 | - | Yes | - | 79.33% | 185 | FRED; FRED Junior |
| 71 | Hanel (69) | 2019 | 2014.7~2015.11 | Prospective study | 141 | 54.6 | - | Yes | - | 87.94% | 141 | PED Classic; PED Flex |
| 72 | Manzato (70) | 2021 | 2016.6~2018.8 | Prospective study | 100 | 52.2 | - | Yes | - | 89.00% | 131 | FRED |
| 73 | Martínez-Galdámez (71) | 2017 | 2015.3~2015.10 | Prospective study | 50 | 53.00 | - | Yes | 54 | 82.00% | 50 | PFED [Pipeline Flex Embolization Device with Shield Technology (Pipeline Shield)] |
| 74 | McAuliffe (72) | 2012 | 2009.8~2010.8 | Prospective study | 54 | 55.7 | 58 | Yes | 98 | 81.48% | 57 | PED |
| 75 | McDougall (73) | 2022 | 2013.7~2016.12 | Prospective study | 145 | 59.1 | 60.1 | No | 155 | 88.97% | 145 | FRED |
| 76 | Petr (74) | 2016 | 2009.6~2015.5 | Prospective study | 310 | 57.40 | - | Yes | - | 80.32% | 318 | PED; Surpass |
| 77 | Raymond (75) | 2022 | 2011.5~2020.11 | Prospective study (RCT) | 278 | ASMO group: 52 FD group: 58 | - | Yes | - | 78.42% | - | - |
| 78 | Salem (76) | 2022 | 2020.2~2021.6 | Retrospective study (Retrospective analysis in prospective databases) | 61 | - | 58 | Yes | - | 81.97% | 72 | FRED; FRED Junior |
| 79 | Wakhloo (77) | 2014 | 2010.4~2013.1 | Prospective study | 161 | 57.10 | - | Yes | 195 | 72.05% | 186 | Surpass |
| 80 | Hanel (78) | 2023 | 2012.10~2015.11 | Prospective study | 180 | 61 | - | No | - | 91.67% | 180 | Surpass (Stryker Neurovascular, Fremont, CA) |
| 81 | Liu (79) | 2018 | 2012.12.18~2014.5.9 | Prospective study (RCT) | 144 | Tubridge group: 52.11 Enterprise group: 55.66 | - | No - | - | 76.39% | Tubridgegroup: 82 Enterprisegroup: 62 | Tubridge; Enterprise |
| 82 | Binh (80) | 2020 | 2012-2017 | Bidirectional Study (Retrospective and prospective) | 130 | - | 50 | Yes | 143 | 76.15% | 134 | PED; FRED; Silk stent |
| 83 | Chalouhi (81) | 2015 | --2013 | Retrospective study | 95 | 53 | - | No | 109 | 89.47% | 95 | PED |
| 84 | Chiu (82) | 2015 | 2009.08-2011.06 | Retrospective study (Retrospective analysis in prospective databases) | 98 | 55.7 | - | Yes | - | 79.59% | 119 | PED |
| 85 | Fischer (83) | 2014 | 2009-2013 | Retrospective study | 65 | - | 62 | No | - | 36.92% | 69 | PED |
| 86 | Fischer (84) | 2011 | 2009.09-2011.01 | Retrospective study (Without informed consent) | 88 | 59 | - | Yes | - | 51.14% | 96 | PED |
| 87 | Gündoğmuş (85) | 2021 | 2012.02-2019.09 | Prospective study | 138 | 51 | - | Yes | 155 | 86.23% | 175 | FRED; PED |
| 88 | Piano (86) | 2021 | 2016.07-2017.09 | Retrospective study | 108 | 56.00 | - | Yes | - | 81.48% | 109 | DERIVO |
| 89 | Piano (87) | 2013 | 2008.10-2011.07 | Retrospective study | 101 | 53 | - | Yes | 120 | 78.22% | 104 | PED; Silk |
| 90 | Xie (88) | 2023 | 2018-2021 | Retrospective study | 57 | 56 | - | Yes | 57 | 66.67% | 77 | Tubridge |
| 91 | Atallah (89) | 2019 | 2011-2016 | Retrospective study | 437 | 52.12 | - | No | - | =- | - | PED |
| 92 | Griessenauer (90) | 2017 | 2009-2015 | Prospective study (Prospective databases) | 117 | - | 54 | Yes | - | 85.47% | 149 | PED |
| 93 | Guo (91) | 2022 | 2016.01-2020.06 | Retrospective study | 114 | Stent-assisted coiling: 56 Flow-diverting devices: 55 | - | Yes | 59 | 57.02% | 114 | PED |
| 94 | Jee (92) | 2021 | SE group: 2019.06-2020.12 Control group: 2014.07-2020.12 | Retrospective study | 84 | 57.5 | - | Yes | 89 | 50.00% | 84 | Surpass Evolve; PED and Surpass Streamline |
| 95 | Kang (93) | 2022 | 2014.11-2019.10 | Retrospective study | 1171 | Only PED (PO): 53.0 PED + coil + incomplete occlusion (PCIO): 54.9 PED + coil + complete occlusion (PCCO): 54.7 | - | Yes | - | 69.37% | 1322 | PED |
| 96 | Khorasanizadeh (94) | 2022 | 2020.06-2021.11 | Retrospective study | 116 | 60.4 | - | Yes | - | 82.76% | 133 | FRED |
| 97 | Kühn (95) | 2019 | 2011.08-2017.08 | Retrospective study | 260 | - | - | - | - | =- | 307 | PED |
| 98 | Luo (96) | 2020 | 2014.11-2019.10 | Retrospective study | 1171 | 53.9 | - | Yes | - | 69.43% | 1322 | PED |
| 99 | Saatci (97) | 2012 | 2008.11-2011.09 | Retrospective study | 191 | 49.2 | - | Yes | 324 | 67.54% | 251 | PED |
| 100 | Salem (98) | 2022 | 2011-2019 | Retrospective study | 95 | - | 57 | Yes | - | 81.05% | 95 | PED; SILK; SURPASS |
| 101 | Sweid (99) | 2018 | 2010-2016 | Retrospective study | 416 | 56.3 | - | Yes | - | 85.35% | 437 | PED |
| 102 | Vranic (100) | 2021 | 2012-2020 | Retrospective study | 189 | Single treatment: 54.0  Retreatment: 59.6 | - | No | - | 89.89% | 189 | PED |
| 103 | Wang (101) | 2022 | 2014.11-2019.10 | Retrospective study | 1171 | 53.9 | - | Yes | - | 69.43% | 1322 | PED classic; PED Flex |
| 104 | Cler (102) | 2022 | 2011-2020 | Prospective study | 222 | - | 56 | Yes | 222 (290) | 82.88% | 290 | PED |

**Supplementary Table S3.** Quality assessment of the observational studies

| NO | Author | Year | Representativenes of the exposed cohort | Selection of non-exposed cohort | Ascertainement of exposure | Demonstration that outcome of interest was not present at start of study | Comparability of cohorts for THE MOST IMPORTANT FACTOR | Comparability of cohorts for THE ADDITIONAL FACTOR | Assessment of outcome | Was follow-up long enough for outcomes to occur | Adequacy of follow-up of cohorts | Total |
| --- | --- | --- | --- | --- | --- | --- | --- | --- | --- | --- | --- | --- |
| 1 | N. Adeeb | 2022 | 1 | 0 | 1 | 0 | 1 | 1 | 1 | 1 | 1 | 7 |
| 2 | J. Berge | 2012 | 1 | 0 | 1 | 0 | 0 | 0 | 1 | 1 | 1 | 5 |
| 3 | P. Bhogal | 2018 | 1 | 0 | 1 | 0 | 0 | 0 | 1 | 1 | 1 | 5 |
| 4 | Bhogal P | 2017 | 1 | 0 | 1 | 0 | 0 | 0 | 1 | 1 | 1 | 5 |
| 5 | Bhogal P | 2016 | 1 | 0 | 1 | 1 | 0 | 0 | 1 | 1 | 1 | 6 |
| 6 | F. Di Maria | 2015 | 1 | 1 | 1 | 0 | 1 | 1 | 1 | 1 | 1 | 8 |
| 7 | Christoph J. Griesse-uer | 2017 | 1 | 0 | 1 | 0 | 0 | 0 | 1 | 1 | 1 | 5 |
| 8 | Christoph J. Griesse-uer | 2018 | 1 | 1 | 1 | 0 | 1 | 1 | 1 | 1 | 1 | 8 |
| 9 | V. Hellstern | 2022 | 1 | 0 | 1 | 0 | 0 | 0 | 1 | 1 | 1 | 5 |
| 10 | Sophia Hohenstatt | 2023 | 1 | 0 | 1 | 0 | 0 | 0 | 1 | 1 | 1 | 5 |
| 11 | Chien-Hui Lee | 2022 | 1 | 0 | 1 | 0 | 0 | 0 | 1 | 1 | 1 | 5 |
| 12 | Ville Nurminen | 2020 | 1 | 0 | 1 | 0 | 0 | 0 | 1 | 1 | 1 | 5 |
| 13 | S. Sirakov | 2019 | 1 | 0 | 1 | 0 | 0 | 0 | 1 | 1 | 1 | 5 |
| 14 | Kathryn M. Wagner | 2019 | 1 | 0 | 1 | 0 | 1 | 1 | 1 | 1 | 0 | 6 |
| 15 | Chao Xu | 2022 | 1 | 1 | 1 | 0 | 1 | 1 | 1 | 1 | 0 | 7 |
| 16 | M. Aguilar Pérez | 2017 | 1 | 0 | 1 | 0 | 0 | 0 | 1 | 1 | 0 | 4 |
| 17 | Marta Aguilar Pérez | 2021 | 1 | 0 | 1 | 0 | 0 | 0 | 1 | 1 | 0 | 4 |
| 18 | Muhammad AlMatter | 2020 | 1 | 0 | 1 | 0 | 0 | 0 | 1 | 1 | 0 | 4 |
| 19 | Alpay K | 2021 | 1 | 0 | 1 | 0 | 0 | 0 | 1 | 1 | 1 | 5 |
| 20 | Leo-rdo B. C. Brasiliense | 2018 | 1 | 0 | 1 | 0 | 1 | 1 | 1 | 1 | 1 | 7 |
| 21 | Francesco Briganti | 2017 | 1 | 0 | 1 | 0 | 0 | 0 | 1 | 1 | 0 | 4 |
| 22 | F. Cag-zzo | 2018 | 1 | 0 | 1 | 0 | 1 | 1 | 1 | 1 | 1 | 7 |
| 23 | Nohra Chalouhi | 2018 | 1 | 0 | 1 | 0 | 0 | 0 | 1 | 1 | 0 | 4 |
| 24 | nohra chalouhi | 2015 | 1 | 0 | 1 | 0 | 0 | 0 | 1 | 1 | 1 | 5 |
| 25 | Tom De Beule | 2020 | 1 | 0 | 1 | 0 | 0 | 0 | 1 | 1 | 1 | 5 |
| 26 | Josser E. Delgado Almandoz | 2017 | 1 | 0 | 1 | 0 | 1 | 1 | 1 | 1 | 1 | 6 |
| 27 | Adam A. Dmytriw | 2019 | 1 | 0 | 1 | 0 | 0 | 0 | 1 | 1 | 0 | 4 |
| 28 | Kareem El -amani | 2023 | 1 | 1 | 1 | 0 | 1 | 1 | 1 | 1 | 0 | 7 |
| 29 | S. Fischer | 2015 | 1 | 0 | 1 | 0 | 0 | 0 | 1 | 1 | 1 | 5 |
| 30 | G Foa Torres | 2018 | 1 | 0 | 1 | 0 | 1 | 1 | 1 | 1 | 1 | 7 |
| 31 | G. Gascou | 2015 | 1 | 0 | 1 | 0 | 0 | 0 | 1 | 1 | 1 | 5 |
| 32 | C. Iosif | 2017 | 1 | 0 | 1 | 0 | 0 | 0 | 1 | 1 | 1 | 5 |
| 33 | Jessica Jesser | 2021 | 1 | 0 | 1 | 0 | 1 | 1 | 1 | 1 | 1 | 7 |
| 34 | Matthew James Kole | 2019 | 1 | 0 | 1 | 0 | 1 | 1 | 1 | 1 | 1 | 7 |
| 35 | Bora Korkmazer | 2019 | 1 | 0 | 1 | 0 | 0 | 0 | 1 | 1 | 0 | 4 |
| 36 | A.L. Kühn | 2020 | 1 | 0 | 1 | 0 | 0 | 0 | 1 | 1 | 1 | 5 |
| 37 | David C Lauzier | 2021 | 1 | 0 | 1 | 0 | 0 | 0 | 1 | 1 | 1 | 5 |
| 38 | Chien-Hui Lee | 2023 | 1 | 0 | 1 | 0 | 0 | 0 | 1 | 1 | 0 | 4 |
| 39 | Li Li | 2022 | 1 | 0 | 1 | 0 | 0 | 0 | 1 | 1 | 1 | 5 |
| 40 | Andreas Simgen | 2021 | 1 | 0 | 1 | 0 | 0 | 0 | 1 | 1 | 1 | 5 |
| 41 | Ivan Lyly | 2021 | 1 | 0 | 1 | 0 | 1 | 1 | 1 | 1 | 1 | 7 |
| 42 | C.J. O’Kelly | 2012 | 1 | 0 | 1 | 0 | 1 | 1 | 1 | 1 | 0 | 6 |
| 43 | A. Poker | 2020 | 1 | 0 | 1 | 0 | 1 | 1 | 1 | 1 | 1 | 7 |
| 44 | José Manuel Pumar | 2016 | 1 | 0 | 1 | 0 | 0 | 0 | 1 | 1 | 0 | 4 |
| 45 | J M Pumar | 2017 | 1 | 0 | 1 | 0 | 0 | 0 | 1 | 1 | 0 | 4 |
| 46 | Mohamed M. Salem | 2020 | 1 | 1 | 1 | 0 | 1 | 1 | 1 | 1 | 1 | 8 |
| 47 | Michael A. Silva | 2018 | 1 | 0 | 1 | 0 | 1 | 1 | 1 | 1 | 1 | 7 |
| 48 | Efe Soydemir | 2023 | 1 | 0 | 1 | 0 | 0 | 0 | 1 | 1 | 1 | 5 |
| 49 | Ido Strauss | 2016 | 1 | 0 | 1 | 0 | 0 | 0 | 1 | 1 | 1 | 5 |
| 50 | Ahmad Sweid | 2020 | 1 | 0 | 1 | 0 | 1 | 1 | 1 | 1 | 1 | 7 |
| 51 | Ahmed K. Toma | 2013 | 1 | 0 | 1 | 0 | 0 | 0 | 1 | 1 | 0 | 4 |
| 52 | Felipe Padovani Trivelato | 2019 | 1 | 0 | 1 | 0 | 1 | 1 | 1 | 1 | 1 | 7 |
| 53 | Vakharia K | 2020 | 1 | 1 | 1 | 0 | 1 | 1 | 1 | 1 | 1 | 8 |
| 54 | Murat Velioglu | 2012 | 1 | 0 | 1 | 0 | 0 | 0 | 1 | 1 | 1 | 5 |
| 55 | Muhammad Waqas | 2020 | 1 | 0 | 1 | 0 | 0 | 0 | 1 | 1 | 1 | 5 |
| 56 | Samer G. Zammar | 2018 | 1 | 0 | 1 | 0 | 1 | 1 | 1 | 1 | 1 | 7 |
| 57 | Qianqian Zhang | 2021 | 1 | 1 | 1 | 0 | 0 | 0 | 1 | 1 | 1 | 6 |
| 58 | Chagas Lourenço G | 2022 | 1 | 0 | 1 | 0 | 1 | 1 | 1 | 1 | 1 | 7 |
| 59 | José E. Cohen | 2021 | 1 | 1 | 1 | 0 | 0 | 0 | 1 | 1 | 1 | 5 |
| 60 | Juan Vivanco-Suarez | 2022 | 1 | 0 | 1 | 0 | 1 | 1 | 1 | 1 | 1 | 7 |
| 61 | F. Drescher | 2017 | 1 | 0 | 1 | 0 | 0 | 0 | 1 | 1 | 0 | 4 |
| 62 | Brasiliense LBC | 2017 | 1 | 0 | 1 | 0 | 1 | 1 | 1 | 1 | 0 | 6 |
| 64 | Martínez-galdámez M | 2019 | 1 | 0 | 1 | 0 | 0 | 0 | 1 | 1 | 0 | 4 |
| 67 | Matthew T. Bender | 2018 | 1 | 0 | 1 | 0 | 1 | 1 | 1 | 1 | 1 | 7 |
| 69 | Jildaz caroff | 2018 | 1 | 1 | 1 | 0 | 0 | 0 | 1 | 1 | 0 | 5 |
| 70 | Guimaraens L | 2020 | 1 | 0 | 1 | 0 | 1 | 1 | 1 | 1 | 0 | 6 |
| 72 | L.B. Manzato | 2021 | 1 | 0 | 1 | 0 | 1 | 1 | 1 | 1 | 1 | 7 |
| 74 | W. McAuliffe | 2012 | 1 | 0 | 1 | 0 | 0 | 0 | 1 | 1 | 1 | 5 |
| 76 | O. Petr | 2016 | 1 | 1 | 1 | 0 | 1 | 0 | 1 | 1 | 1 | 7 |
| 78 | Mohamed M. Salem | 2022 | 1 | 0 | 1 | 0 | 0 | 0 | 1 | 1 | 1 | 5 |
| 79 | A.K. Wakhloo | 2014 | 1 | 0 | 1 | 0 | 0 | 0 | 1 | 1 | 1 | 5 |
| 82 | Nguyen Thai Binh | 2020 | 1 | 0 | 1 | 0 | 0 | 0 | 1 | 1 | 0 | 4 |
| 83 | Nohra Chalouhi | 2015 | 1 | 0 | 1 | 0 | 1 | 1 | 1 | 1 | 0 | 6 |
| 84 | A.H.Y. Chiu | 2015 | 1 | 0 | 1 | 0 | 0 | 0 | 1 | 1 | 1 | 5 |
| 85 | Sebastian Fischer | 2014 | 1 | 0 | 1 | 0 | 1 | 1 | 1 | 1 | 1 | 7 |
| 86 | Sebastian Fischer | 2011 | 1 | 0 | 1 | 0 | 0 | 0 | 1 | 1 | 1 | 5 |
| 87 | Cemal A Gündoğ muş | 2021 | 1 | 1 | 1 | 0 | 1 | 1 | 1 | 1 | 1 | 8 |
| 88 | Mariangela PiaNo | 2021 | 1 | 0 | 1 | 0 | 0 | 0 | 1 | 1 | 1 | 5 |
| 89 | MARIANGELA PIANO | 2013 | 1 | 0 | 1 | 0 | 0 | 0 | 1 | 1 | 1 | 5 |
| 90 | Dajiang Xie | 2023 | 1 | 0 | 1 | 0 | 0 | 0 | 1 | 1 | 1 | 5 |
| 91 | Elias Atallah | 2019 | 1 | 0 | 1 | 0 | 1 | 1 | 1 | 1 | 1 | 7 |
| 92 | Christoph J. Griesse-uer | 2017 | 1 | 0 | 1 | 0 | 1 | 1 | 1 | 1 | 1 | 7 |
| 93 | Hao Guo | 2022 | 1 | 1 | 1 | 0 | 1 | 1 | 1 | 1 | 0 | 7 |
| 94 | Tae Keun Jee | 2021 | 1 | 1 | 1 | 0 | 1 | 1 | 1 | 1 | 0 | 7 |
| 95 | Huibin Kang | 2022 | 1 | 1 | 1 | 0 | 1 | 1 | 1 | 1 | 0 | 7 |
| 96 | MirHojjat Khorasanizadeh | 2022 | 1 | 0 | 1 | 0 | 1 | 1 | 1 | 1 | 1 | 7 |
| 97 | An- Luisa Kühn | 2019 | 1 | 0 | 1 | 0 | 1 | 1 | 1 | 1 | 1 | 7 |
| 98 | Bin Luo | 2020 | 1 | 1 | 1 | 0 | 1 | 1 | 1 | 1 | 0 | 7 |
| 99 | I. Saatci | 2012 | 1 | 0 | 1 | 0 | 0 | 0 | 1 | 1 | 1 | 5 |
| 100 | Mohamed M. Salem | 2022 | 1 | 0 | 1 | 0 | 0 | 0 | 1 | 1 | 1 | 5 |
| 101 | A. Sweid | 2018 | 1 | 1 | 1 | 0 | 1 | 1 | 1 | 1 | 1 | 8 |
| 102 | Justin E Vranic | 2021 | 1 | 0 | 1 | 0 | 1 | 1 | 1 | 1 | 1 | 7 |
| 103 | Chao Wang | 2022 | 1 | 1 | 1 | 0 | 1 | 1 | 1 | 1 | 0 | 7 |
| 104 | Samuel J. Cler | 2022 | 1 | 0 | 1 | 0 | 1 | 1 | 1 | 1 | 1 | 7 |

**Supplementary Table S4.** Quality assessment of the single-arm clinical trials

| No. | Author | Year | A clearly stated aim | Inclusion of consecutive patients | Prospective collection of data | Endpoints appropriate to the aim of the study | Unbiased assessment of the study endpoint | Follow-up period appropriate to the aim of the study | Loss to follow up less than 5% | Prospective calculation of the study size | Total |
| --- | --- | --- | --- | --- | --- | --- | --- | --- | --- | --- | --- |
| 5 | Hanel RA | 2023 | 2 | 0 | 2 | 1 | 0 | 2 | 2 | 0 | 9 |
| 11 | Philip M. Meyers | 2019 | 2 | 2 | 2 | 2 | 0 | 2 | 2 | 0 | 12 |
| 12 | Felipe Padovani Trivelato | 2019 | 2 | 0 | 2 | 1 | 0 | 2 | 2 | 0 | 10 |
| 14 | Alain Bo-fe | 2022 | 2 | 2 | 2 | 1 | 0 | 2 | 2 | 1 | 12 |
| 23 | Hanel RA | 2019 | 2 | 0 | 2 | 1 | 0 | 2 | 2 | 2 | 11 |
| 27 | Mario Martínez-Galdámez | 2017 | 2 | 0 | 2 | 1 | 0 | 1 | 2 | 0 | 8 |
| 30 | McDougall CG | 2022 | 2 | 0 | 2 | 2 | 0 | 2 | 0 | 0 | 8 |
| 49 | Ricardo A. Hanel | 2022 | 2 | 2 | 2 | 2 | 0 | 2 | 0 | 0 | 10 |

**Supplementary Table S5.** Publication bias

| Outcomes | *P* |
| --- | --- |
| Complete/near complete occlusion rate at 6 months after the procedure | 0.772 |
| Complete/near complete occlusion rate at 12 months after the procedure | 0.771 |
| Complete/near complete occlusion rate at 24 months after the procedure | 0.752 |
| Complete/near complete occlusion rate at 36 months after the procedure | 0.304 |
| Complete occlusion at 6 months after the procedure | 0.226 |
| Complete occlusion at 12 months after the procedure | 0.680 |
| Complete occlusion at 24 months after the procedure | 0.329 |
| Complete occlusion at 36 months after the procedure | 0.025 |
| Total postoperative rescue therapy rate | 0.425 |
| Rescue therapy rate at 1 year after the procedure | 0.672 |
| mRS score ≤ 2 at 1 month after the procedure | 0.708 |
| mRS score ≤ 2 at 3 months after the procedure | 0.415 |
| mRS score ≤ 2 at 6 months after the procedure | 0.365 |
| mRS score ≤ 2 at 1 year after the procedure | 0.170 |
| Thromboembolic event with unspecified follow-up duration | 0.743 |
| Hemorrhagic event with unspecified follow-up duration | <0.001 |
| Ischemic event with unspecified follow-up duration | <0.001 |
| Thromboembolic event at 1 month after the procedure | 0.391 |
| Hemorrhagic event at 1 month after the procedure | 0.384 |
| Ischemic event at 1 month after the procedure | 0.002 |
| Neurogenic death at 1 month after the procedure | 0.047 |
| Incidence of ISR at 6 months after the procedure | 0.169 |
| Incidence of ISR at 12 months after the procedure | 0.760 |
| Incidence of total ISR | <0.001 |
| Treatment-related complications | 0.002 |
| Aneurysm rupture | 0.292 |
| Procedural success rate | 0.097 |
| All-cause mortality | 0.662 |

Supplementary Figure S1. Funnel plots for publication bias assessment. (A–D) Complete/near-complete occlusion rate at 6, 12, 24 and 36 months post-procedure, respectively. (E–H) Complete occlusion rate at 6, 12, 24 and 36 months post-procedure, respectively. (I) Total postoperative rescue therapy rate. (J) Rescue therapy rate at 1 year post-procedure. (K–N) Rate of mRS score ≤2 at 1 month, 3 months, 6 months and 1 year post-procedure, respectively.

Supplementary Figure S2. Funnel plots for publication bias assessment of complication and outcome rates. (A–C) Thromboembolic, hemorrhagic, and ischemic events with unspecified follow-up duration, respectively. (D–F) Thromboembolic, hemorrhagic, and ischemic events at 1 month post-procedure, respectively. (G) Neurogenic death at 1 month post-procedure. (H–J) Incidence of in-stent restenosis (ISR) at 6 months, 12 months, and overall, respectively. (K) Treatment-related complications. (L) Aneurysm rupture. (M) Procedural success rate. (N) All-cause mortality.

**References**

1.	Adeeb N, Dibas M, Griessenauer CJ, Cuellar HH, Salem MM, Xiang S, et al. Learning Curve for Flow Diversion of Posterior Circulation Aneurysms: A Long-Term International Multicenter Cohort Study. AJNR Am J Neuroradiol (2022) 43:1615-20.

2.	Berge J, Biondi A, Machi P, Brunel H, Pierot L, Gabrillargues J, et al. Flow-diverter silk stent for the treatment of intracranial aneurysms: 1-year follow-up in a multicenter study. AJNR Am J Neuroradiol (2012) 33:1150-5.

3.	Bhogal P, Chudyk J, Bleise C, Lylyk I, Perez N, Henkes H, et al. Treatment of Unruptured, Tandem Aneurysms of the ICA with a Single Flow Diverter. Clin Neuroradiol (2019) 29:725-31.

4.	Bhogal P, Perez MA, Ganslandt O, Bazner H, Henkes H, Fischer S. Treatment of posterior circulation non-saccular aneurysms with flow diverters: a single-center experience and review of 56 patients. J Neurointerv Surg (2017) 9:471-81.

5.	Di Maria F, Pistocchi S, Clarencon F, Bartolini B, Blanc R, Biondi A, et al. Flow Diversion versus Standard Endovascular Techniques for the Treatment of Unruptured Carotid-Ophthalmic Aneurysms. AJNR Am J Neuroradiol (2015) 36:2325-30.

6.	Griessenauer CJ, Piske RL, Baccin CE, Pereira BJA, Reddy AS, Thomas AJ, et al. Flow Diverters for Treatment of 160 Ophthalmic Segment Aneurysms: Evaluation of Safety and Efficacy in a Multicenter Cohort. Neurosurgery (2017) 80:726-32.

7.	Griessenauer CJ, Ogilvy CS, Adeeb N, Dmytriw AA, Foreman PM, Shallwani H, et al. Pipeline embolization of posterior circulation aneurysms: a multicenter study of 131 aneurysms. J Neurosurg (2018) 130:923-35.

8.	Hellstern V, Aguilar Perez M, Henkes E, Donauer E, Wendl C, Bazner H, et al. Use of a p64 MW Flow Diverter with Hydrophilic Polymer Coating (HPC) and Prasugrel Single Antiplatelet Therapy for the Treatment of Unruptured Anterior Circulation Aneurysms: Safety Data and Short-term Occlusion Rates. Cardiovasc Intervent Radiol (2022) 45:1364-74.

9.	Hohenstatt S, Vinci SL, Vollherbst DF, Tessitore A, Schmitt N, Pitrone A, et al. Flow Diverting Stents in Cerebral Small Caliber Vessels (< 2 mm) for Aneurysm Treatment : A Three Center Retrospective Study. Clin Neuroradiol (2023) 33:99-105.

10.	Lee CH, Luo CB, Lai YC, Chang FC, Lin CJ. Flow diverter manages very small aneurysm of the internal carotid artery. J Chin Med Assoc (2022) 85:754-8.

11.	Nurminen V, Raj R, Numminen J, Kivisaari R, Niemela M, Lehecka M. Flow diversion for internal carotid artery aneurysms: Impact of complex aneurysm features and overview of outcome. Clin Neurol Neurosurg (2020) 193:105782.

12.	Sirakov S, Sirakov A, Bhogal P, Penkov M, Minkin K, Ninov K, et al. The p64 Flow Diverter-Mid-term and Long-term Results from a Single Center. Clin Neuroradiol (2020) 30:471-80.

13.	Wagner KM, Srinivasan VM, Srivatsan A, Ghali MGZ, Thomas AJ, Enriquez-Marulanda A, et al. Outcomes after coverage of lenticulostriate vessels by flow diverters: a multicenter experience. J Neurosurg (2019) 132:473-80.

14.	Xu C, Wu P, Sun B, Xu S, Luo B, Yang X, et al. Incomplete occlusion and visual symptoms of peri-ophthalmic aneurysm after treatment with a pipeline embolization device: a multi-center cohort study. Acta Neurochir (Wien) (2022) 164:2191-202.

15.	Aguilar Perez M, Bhogal P, Henkes E, Ganslandt O, Bazner H, Henkes H. In-stent Stenosis after p64 Flow Diverter Treatment. Clin Neuroradiol (2018) 28:563-8.

16.	Aguilar Perez M, Henkes E, Hellstern V, Serna Candel C, Wendl C, Bazner H, et al. Endovascular Treatment of Anterior Circulation Aneurysms With the p64 Flow Modulation Device: Mid- and Long-Term Results in 617 Aneurysms From a Single Center. Oper Neurosurg (Hagerstown) (2021) 20:355-63.

17.	AlMatter M, Henkes E, Sirakov A, Aguilar Perez M, Hellstern V, Serna Candel C, et al. The p48 MW flow modulation device for treatment of unruptured, saccular intracranial aneurysms: a single center experience from 77 consecutive aneurysms. CVIR Endovasc (2020) 3:39.

18.	Alpay K, Hinkka T, Lindgren AE, Isokangas JM, Raj R, Parkkola R, et al. Finnish flow diverter study: 8 years of experience in the treatment of acutely ruptured intracranial aneurysms. J Neurointerv Surg (2022) 14:699-703.

19.	Brasiliense LBC, Aguilar-Salinas P, Lopes DK, Nogueira D, DeSousa K, Nelson PK, et al. Multicenter Study of Pipeline Flex for Intracranial Aneurysms. Neurosurgery (2019) 84:E402-E9.

20.	Briganti F, Leone G, Cirillo L, de Divitiis O, Solari D, Cappabianca P. Postprocedural, midterm, and long-term results of cerebral aneurysms treated with flow-diverter devices: 7-year experience at a single center. Neurosurg Focus (2017) 42:E3.

21.	Cagnazzo F, Cappucci M, Dargazanli C, Lefevre PH, Gascou G, Riquelme C, et al. Flow-Diversion Effect of LEO Stents: Aneurysm Occlusion and Flow Remodeling of Covered Side Branches and Perforators. AJNR Am J Neuroradiol (2018) 39:2057-63.

22.	Chalouhi N, Patel PD, Atallah E, Starke RM, Chitale A, Lang M, et al. Low Yield of Cerebral Angiography in Adequately Occluded Aneurysms After Flow Diversion. Neurosurgery (2018) 83:1294-7.

23.	Chalouhi N, Zanaty M, Whiting A, Yang S, Tjoumakaris S, Hasan D, et al. Safety and efficacy of the Pipeline Embolization Device in 100 small intracranial aneurysms. J Neurosurg (2015) 122:1498-502.

24.	De Beule T, Boulanger T, Heye S, van Rooij WJ, van Zwam WH, Stockx L. p64 flow diverter: Results in 108 patients from a single center. Interv Neuroradiol (2021) 27:51-9.

25.	Delgado Almandoz JE, Kayan Y, Tenreiro A, Wallace AN, Scholz JM, Fease JL, et al. Clinical and angiographic outcomes in patients with intracranial aneurysms treated with the pipeline embolization device: intra-procedural technical difficulties, major morbidity, and neurological mortality decrease significantly with increased operator experience in device deployment and patient management. Neuroradiology (2017) 59:1291-9.

26.	Dmytriw AA, Phan K, Moore JM, Pereira VM, Krings T, Thomas AJ. On Flow Diversion: The Changing Landscape of Intracerebral Aneurysm Management. AJNR Am J Neuroradiol (2019) 40:591-600.

27.	El Naamani K, Saad H, Chen CJ, Abbas R, Sioutas GS, Amllay A, et al. Comparison of Flow-Redirection Endoluminal Device and Pipeline Embolization Device in the Treatment of Intracerebral Aneurysms. Neurosurgery (2023) 92:118-24.

28.	Fischer S, Aguilar-Perez M, Henkes E, Kurre W, Ganslandt O, Bazner H, et al. Initial Experience with p64: A Novel Mechanically Detachable Flow Diverter for the Treatment of Intracranial Saccular Sidewall Aneurysms. AJNR Am J Neuroradiol (2015) 36:2082-9.

29.	Foa Torres G, Roca F, Noguera A, Godes J, Petrocelli S, Aznar I, et al. Silk flow-diverter stent for the treatment of complex intracranial aneurysms: A one-year follow-up multicenter study. Interv Neuroradiol (2018) 24:357-62.

30.	Gascou G, Lobotesis K, Brunel H, Machi P, Riquelme C, Eker O, et al. Extra-aneurysmal flow modification following pipeline embolization device implantation: focus on regional branches, perforators, and the parent vessel. AJNR Am J Neuroradiol (2015) 36:725-31.

31.	Iosif C, Mounayer C, Yavuz K, Saleme S, Geyik S, Cekirge HS, et al. Middle Cerebral Artery Bifurcation Aneurysms Treated by Extrasaccular Flow Diverters: Midterm Angiographic Evolution and Clinical Outcome. AJNR Am J Neuroradiol (2017) 38:310-6.

32.	Jesser J, Alberalar ND, Kizilkilic O, Saatci I, Baltacioglu F, Ozluk E, et al. Safety and Efficacy of the FRED Jr Flow Re-Direction Endoluminal Device for Intracranial Aneurysms: Retrospective Multicenter Experience With Emphasis on Midterm Results. Front Neurol (2021) 12:722183.

33.	Kole MJ, Miller TR, Cannarsa G, Wessell A, Jones S, Le E, et al. Pipeline embolization device diameter is an important factor determining the efficacy of flow diversion treatment of small intracranial saccular aneurysms. J Neurointerv Surg (2019) 11:1004-8.

34.	Korkmazer B, Kocak B, Islak C, Kocer N, Kizilkilic O. Long-term results of flow diversion in the treatment of intracranial aneurysms: a retrospective data analysis of a single center. Acta Neurochir (Wien) (2019) 161:1165-73.

35.	Kuhn AL, Gounis MJ, Puri AS. Introduction: History and Development of Flow Diverter Technology and Evolution. Neurosurgery (2020) 86:S3-S10.

36.	Lauzier DC, Cler SJ, Chatterjee AR, Osbun JW, Moran CJ, Kansagra AP. The value of long-term angiographic follow-up following Pipeline embolization of intracranial aneurysms. J Neurointerv Surg (2022) 14:585-8.

37.	Li L, Gao BL, Shao QJ, Zhang GL, Wang ZL, Li TX, et al. Small Unruptured Intracranial Aneurysms Can Be Effectively Treated With Flow-Diverting Devices. Front Neurol (2022) 13:913653.

38.	Simgen A, Mayer C, Kettner M, Muhl-Benninghaus R, Reith W, Yilmaz U. Retrospective analysis of intracranial aneurysms after flow diverter treatment including color-coded imaging (syngo iFlow) as a predictor of aneurysm occlusion. Interv Neuroradiol (2022) 28:190-200.

39.	Lylyk I, Scrivano E, Lundquist J, Ferrario A, Bleise C, Perez N, et al. Pipeline Embolization Devices for the Treatment of Intracranial Aneurysms, Single-Center Registry: Long-Term Angiographic and Clinical Outcomes from 1000 Aneurysms. Neurosurgery (2021) 89:443-9.

40.	O'Kelly CJ, Spears J, Chow M, Wong J, Boulton M, Weill A, et al. Canadian experience with the pipeline embolization device for repair of unruptured intracranial aneurysms. AJNR Am J Neuroradiol (2013) 34:381-7.

41.	Poker A, Ocal O, Ozturk E, Arat A. Propensity Score Analysis of Flow Diverters Placed in Scaffolding Stents. AJNR Am J Neuroradiol (2021) 42:1093-8.

42.	Pumar JM, Banguero A, Cuellar H, Guimaraens L, Masso J, Miralbes S, et al. Treatment of Intracranial Aneurysms With the SILK Embolization Device in a Multicenter Study. A Retrospective Data Analysis. Neurosurgery (2017) 81:595-601.

43.	Pumar JM, Mosqueira A, Cuellar H, Dieguez B, Guimaraens L, Masso J, et al. Expanding the use of flow diverters beyond their initial indication: treatment of small unruptured aneurysms. J Neurointerv Surg (2018) 10:245-8.

44.	Salem MM, Ravindran K, Enriquez-Marulanda A, Ascanio LC, Jordan N, Gomez-Paz S, et al. Pipeline Embolization Device Versus Stent-Assisted Coiling for Intracranial Aneurysm Treatment: A Retrospective Propensity Score-Matched Study. Neurosurgery (2020) 87:516-22.

45.	Silva MA, See AP, Khandelwal P, Mahapatra A, Frerichs KU, Du R, et al. Comparison of flow diversion with clipping and coiling for the treatment of paraclinoid aneurysms in 115 patients. J Neurosurg (2018):1-8.

46.	Soydemir E, Gundogmus CA, Tureli D, Andac Baltacioglu N, Bayri Y, Baltacioglu F. Safety and efficacy of flow diverter stents in the treatment of middle cerebral artery aneurysms: a single-center experience and follow-up data. Diagn Interv Radiol (2023) 29:350-8.

47.	Strauss I, Maimon S. Silk flow diverter in the treatment of complex intracranial aneurysms: a single-center experience with 60 patients. Acta Neurochir (Wien) (2016) 158:247-54.

48.	Sweid A, Starke RM, Herial N, Chalouhi N, Das S, Baldassari MP, et al. Predictors of Complications, Functional Outcome, and Morbidity in a Large Cohort Treated With Flow Diversion. Neurosurgery (2020) 87:730-43.

49.	Toma AK, Robertson F, Wong K, Joshi Y, Haridas A, Grieve J, et al. Early single centre experience of flow diverting stents for the treatment of cerebral aneurysms. Br J Neurosurg (2013) 27:622-8.

50.	Trivelato FP, Ulhoa AC, Rezende MT, Castro-Afonso LH, Abud DG. Republished: Recurrence of a totally occluded aneurysm after treatment with a pipeline embolization device. J Neurointerv Surg (2019) 11:e5.

51.	Vakharia K, Munich SA, Waqas M, Levy EI, Siddiqui AH. Treatment of Anterior Circulation Aneurysms in the Internal Carotid Artery With Flow Diverters. Neurosurgery (2020) 86:S55-S63.

52.	Velioglu M, Kizilkilic O, Selcuk H, Kocak B, Tureci E, Islak C, et al. Early and midterm results of complex cerebral aneurysms treated with Silk stent. Neuroradiology (2012) 54:1355-65.

53.	Waqas M, Vakharia K, Gong AD, Rai HH, Wack A, Fayyaz N, et al. One and done? The effect of number of Pipeline embolization devices on aneurysm treatment outcomes. Interv Neuroradiol (2020) 26:147-55.

54.	Zammar SG, Buell TJ, Chen CJ, Crowley RW, Ding D, Griessenauer CJ, et al. Outcomes After Off-Label Use of the Pipeline Embolization Device for Intracranial Aneurysms: A Multicenter Cohort Study. World Neurosurg (2018) 115:e200-e5.

55.	Zhang Q, Shao Q, Chang K, Zhang H, He Y, Andrade-Barazarte H, et al. Safety and Efficacy of Coils in Conjunction With the Pipeline Flex Embolization Device for the Treatment of Cerebral Aneurysms. Front Neurol (2021) 12:651465.

56.	Chagas Lourenco G, Dantas F, Silva ECT, Firmino RUR, Quesado Filgueiras Filho M, Tosello RT, et al. Endovascular treatment of intracranial aneurysms using the Derivo Embolization Device: a multicenter experience. J Neurointerv Surg (2023) 15:776-80.

57.	Cohen JE, Gomori JM, Moscovici S, Kaye AH, Shoshan Y, Spektor S, et al. Flow-diverter stents in the early management of acutely ruptured brain aneurysms: effective rebleeding protection with low thromboembolic complications. J Neurosurg (2021):1-8.

58.	Vivanco-Suarez J, Mendez-Ruiz A, Farooqui M, Bekelis K, Singer JA, Javed K, et al. Safety and efficacy of the surpass streamline for intracranial aneurysms (SESSIA): A multi-center US experience pooled analysis. Interv Neuroradiol (2023) 29:589-98.

59.	Drescher F, Weber W, Berlis A, Rohde S, Carolus A, Fischer S. Treatment of Intra- and Extracranial Aneurysms Using the Flow-Redirection Endoluminal Device: Multicenter Experience and Follow-Up Results. AJNR Am J Neuroradiol (2017) 38:105-12.

60.	Brasiliense LBC, Aguilar-Salinas P, Miller DA, Tawk RG, Sauvageau EA, Hanel RA. Analysis of Predictors and Probability of Aneurysm Occlusion in the Internal Carotid Artery After Treatment with Pipeline Embolization Device. World Neurosurg (2017) 107:641-8.

61.	Hanel RA, Cortez GM, Lopes DK, Nelson PK, Siddiqui AH, Jabbour P, et al. Prospective study on embolization of intracranial aneurysms with the pipeline device (PREMIER study): 3-year results with the application of a flow diverter specific occlusion classification. J Neurointerv Surg (2023) 15:248-54.

62.	Martinez-Galdamez M, Lamin SM, Lagios KG, Liebig T, Ciceri EF, Chapot R, et al. Treatment of intracranial aneurysms using the pipeline flex embolization device with shield technology: angiographic and safety outcomes at 1-year follow-up. J Neurointerv Surg (2019) 11:396-9.

63.	Meyers PM, Coon AL, Kan PT, Wakhloo AK, Hanel RA. SCENT Trial. Stroke (2019) 50:1473-9.

64.	Trivelato FP, Abud DG, Ulhoa AC, Waihrich ES, Abud TG, Castro Afonso LH, et al. Derivo Embolization Device for the Treatment of Intracranial Aneurysms. Stroke (2019) 50:2351-8.

65.	Bender MT, Vo CD, Jiang B, Campos JK, Zarrin DA, Xu R, et al. Pipeline Embolization for Salvage Treatment of Previously Stented Residual and Recurrent Cerebral Aneurysms. Interv Neurol (2018) 7:359-69.

66.	Bonafe A, Perez MA, Henkes H, Lylyk P, Bleise C, Gascou G, et al. Diversion-p64: results from an international, prospective, multicenter, single-arm post-market study to assess the safety and effectiveness of the p64 flow modulation device. J Neurointerv Surg (2022) 14:898-903.

67.	Caroff J, Iacobucci M, Rouchaud A, Mihalea C, de Carvalho FM, Jocson VED, et al. The occurrence of neointimal hyperplasia after flow-diverter implantation is associated with cardiovascular risks factors and the stent design. J Neurointerv Surg (2019) 11:610-3.

68.	Guimaraens L, Vivas E, Saldana J, Llibre JC, Gil A, Balaguer E, et al. Efficacy and safety of the dual-layer flow-diverting stent (FRED) for the treatment of intracranial aneurysms. J Neurointerv Surg (2020) 12:521-5.

69.	Hanel RA, Kallmes DF, Lopes DK, Nelson PK, Siddiqui A, Jabbour P, et al. Prospective study on embolization of intracranial aneurysms with the pipeline device: the PREMIER study 1 year results. J Neurointerv Surg (2020) 12:62-6.

70.	Manzato LB, Santos RB, Filho PMM, Miotto G, Bastos AM, Vanzin JR. Brazilian FRED Registry: A Prospective Multicenter Study for Brain Aneurysm Treatment-The BRED Study. AJNR Am J Neuroradiol (2021) 42:1822-6.

71.	Martinez-Galdamez M, Lamin SM, Lagios KG, Liebig T, Ciceri EF, Chapot R, et al. Periprocedural outcomes and early safety with the use of the Pipeline Flex Embolization Device with Shield Technology for unruptured intracranial aneurysms: preliminary results from a prospective clinical study. J Neurointerv Surg (2017) 9:772-6.

72.	McAuliffe W, Wycoco V, Rice H, Phatouros C, Singh TJ, Wenderoth J. Immediate and midterm results following treatment of unruptured intracranial aneurysms with the pipeline embolization device. AJNR Am J Neuroradiol (2012) 33:164-70.

73.	McDougall CG, Diaz O, Boulos A, Siddiqui AH, Caplan J, Fifi JT, et al. Safety and efficacy results of the Flow Redirection Endoluminal Device (FRED) stent system in the treatment of intracranial aneurysms: US pivotal trial. J Neurointerv Surg (2022) 14:577-84.

74.	Petr O, Brinjikji W, Cloft H, Kallmes DF, Lanzino G. Current Trends and Results of Endovascular Treatment of Unruptured Intracranial Aneurysms at a Single Institution in the Flow-Diverter Era. AJNR Am J Neuroradiol (2016) 37:1106-13.

75.	Raymond J, Iancu D, Boisseau W, Diestro JDB, Klink R, Chagnon M, et al. Flow Diversion in the Treatment of Intracranial Aneurysms: A Pragmatic Randomized Care Trial. AJNR Am J Neuroradiol (2022) 43:1244-51.

76.	Salem MM, Kvint S, Hendrix P, Al Saiegh F, Gajjar AA, Choudhri O, et al. The Pennsylvania Postmarket Multicenter Experience With Flow Redirection Endoluminal Device. Neurosurgery (2022) 91:280-5.

77.	Wakhloo AK, Lylyk P, de Vries J, Taschner C, Lundquist J, Biondi A, et al. Surpass flow diverter in the treatment of intracranial aneurysms: a prospective multicenter study. AJNR Am J Neuroradiol (2015) 36:98-107.

78.	Hanel RA, Cortez GM, Coon AL, Kan P, Taussky P, Wakhloo AK, et al. Surpass Intracranial Aneurysm Embolization System Pivotal Trial to Treat Large or Giant Wide-Neck Aneurysms - SCENT: 3-year outcomes. J Neurointerv Surg (2023) 15:1084-9.

79.	Liu JM, Zhou Y, Li Y, Li T, Leng B, Zhang P, et al. Parent Artery Reconstruction for Large or Giant Cerebral Aneurysms Using the Tubridge Flow Diverter: A Multicenter, Randomized, Controlled Clinical Trial (PARAT). AJNR Am J Neuroradiol (2018) 39:807-16.

80.	Binh NT, Luu VD, Thong PM, Cuong NN, Anh NQ, Tuan TA, et al. Flow diverter stent for treatment of cerebral aneurysms: A report of 130 patients with 134 aneurysms. Heliyon (2020) 6:e03356.

81.	Chalouhi N, Zanaty M, Whiting A, Tjoumakaris S, Hasan D, Ajiboye N, et al. Treatment of ruptured intracranial aneurysms with the pipeline embolization device. Neurosurgery (2015) 76:165-72; discussion 72.

82.	Chiu AH, Cheung AK, Wenderoth JD, De Villiers L, Rice H, Phatouros CC, et al. Long-Term Follow-Up Results following Elective Treatment of Unruptured Intracranial Aneurysms with the Pipeline Embolization Device. AJNR Am J Neuroradiol (2015) 36:1728-34.

83.	Fischer S, Perez MA, Kurre W, Albes G, Bazner H, Henkes H. Pipeline embolization device for the treatment of intra- and extracranial fusiform and dissecting aneurysms: initial experience and long-term follow-up. Neurosurgery (2014) 75:364-74; discussion 74.

84.	Fischer S, Brunk BP, Chen F, Gao X, Harb OS, Iodice JB, et al. Using OrthoMCL to assign proteins to OrthoMCL-DB groups or to cluster proteomes into new ortholog groups. Curr Protoc Bioinformatics (2011) Chapter 6:6 12 1-6 9.

85.	Gundogmus CA, Sabet S, Baltacioglu NA, Tureli D, Bayri Y, Baltacioglu F. Long-term results and comparison of flow re-direction endoluminal device and pipeline embolization device in endovascular treatment of intracranial carotid aneurysms. Interv Neuroradiol (2022) 28:302-10.

86.	Piano M, Lozupone E, Sgoifo A, Nuzzi NP, Asteggiano F, Pero G, et al. Long-term follow-up of the Derivo(R) Embolization Device (DED(R)) for intracranial aneurysms: the Italian Multicentric Registry. J Neurosurg Sci (2021) 65:361-8.

87.	Piano M, Valvassori L, Quilici L, Pero G, Boccardi E. Midterm and long-term follow-up of cerebral aneurysms treated with flow diverter devices: a single-center experience. J Neurosurg (2013) 118:408-16.

88.	Xie D, Yang H, Zhao L, Ye X, Yang S, Gao C, et al. Tubridge flow diverter for the treatment of small and medium aneurysms. Front Neurol (2023) 14:1054631.

89.	Atallah E, Saad H, Mouchtouris N, Bekelis K, Walker J, Chalouhi N, et al. Pipeline for Distal Cerebral Circulation Aneurysms. Neurosurgery (2019) 85:E477-E84.

90.	Griessenauer CJ, Ogilvy CS, Foreman PM, Chua MH, Harrigan MR, He L, et al. Pipeline Embolization Device for Small Intracranial Aneurysms: Evaluation of Safety and Efficacy in a Multicenter Cohort. Neurosurgery (2017) 80:579-87.

91.	Guo H, Liu JF, Li CH, Wang JW, Li H, Gao BL. Effects of stent-assisted coiling in comparison with flow diversion on intracranial aneurysms. Front Neurol (2022) 13:937536.

92.	Jee TK, Yeon JY, Kim KH, Kim JS, Hong SC, Jeon P. Early clinical experience of using the Surpass Evolve flow diverter in the treatment of intracranial aneurysms. Neuroradiology (2022) 64:343-51.

93.	Kang H, Luo B, Liu J, Zhang H, Li T, Song D, et al. Postoperative occlusion degree after flow-diverter placement with adjunctive coiling: analysis of complications. J Neurointerv Surg (2022) 14:371-5.

94.	Khorasanizadeh M, Shutran M, Schirmer CM, Salem MM, Ringer AJ, Grandhi R, et al. North American multicenter experience with the Flow Redirection Endoluminal Device in the treatment of intracranial aneurysms. J Neurosurg (2023) 138:933-43.

95.	Kuhn AL, Kan P, Srinivasan V, Rex DE, de Macedo Rodrigues K, Howk MC, et al. Flow diverter for endovascular treatment of intracranial mirror segment internal carotid artery aneurysms. Interv Neuroradiol (2019) 25:4-11.

96.	Luo B, Kang H, Zhang H, Li T, Liu J, Song D, et al. Pipeline Embolization device for intracranial aneurysms in a large Chinese cohort: factors related to aneurysm occlusion. Ther Adv Neurol Disord (2020) 13:1756286420967828.

97.	Saatci I, Yavuz K, Ozer C, Geyik S, Cekirge HS. Treatment of intracranial aneurysms using the pipeline flow-diverter embolization device: a single-center experience with long-term follow-up results. AJNR Am J Neuroradiol (2012) 33:1436-46.

98.	Salem MM, Sweid A, Kuhn AL, Dmytriw AA, Gomez-Paz S, Maragkos GA, et al. Repeat Flow Diversion for Cerebral Aneurysms Failing Prior Flow Diversion: Safety and Feasibility From Multicenter Experience. Stroke (2022) 53:1178-89.

99.	Sweid A, Atallah E, Herial N, Saad H, Mouchtouris N, Barros G, et al. Pipeline-assisted coiling versus pipeline in flow diversion treatment of intracranial aneurysms. J Clin Neurosci (2018) 58:20-4.

100.	Vranic JE, Harker P, Stapleton CJ, Regenhardt RW, Dmytriw AA, Alotaibi NM, et al. Determinants of intracranial aneurysm retreatment following embolization with a single flow-diverting stent. Neuroradiol J (2022) 35:461-7.

101.	Wang C, Luo B, Li T, Maimaitili A, Mao G, Song D, et al. Comparison of the Pipeline embolisation device alone or combined with coiling for treatment of different sizes of intracranial aneurysms. Stroke Vasc Neurol (2022) 7:345-52.

102.	Cler SJ, Lauzier DC, Chatterjee AR, Osbun JW, Moran CJ, Kansagra AP. Time Line of Occlusion for Intracranial Aneurysms Treated with the Pipeline Embolization Device. World Neurosurg (2022) 166:e750-e7.
